# Association of gut microbiota with post-operative clinical course in Crohn’s disease

**DOI:** 10.1186/1471-230X-13-131

**Published:** 2013-08-22

**Authors:** Neelendu Dey, David AW Soergel, Susanna Repo, Steven E Brenner

**Affiliations:** 1 Department of Medicine, Division of Gastroenterology, University of California, San Francisco, 513 Parnassus Avenue, Room S-357, San Francisco, CA 94143-0538, USA; 2 Department of Plant and Microbial Biology, University of California, Berkeley, 461 Koshland Hall, Berkeley, CA 94720-3102, USA; 3 Current address: Center for Genome Sciences and Systems Biology, Washington University School of Medicine, Saint Louis, MO 63108, USA; 4 Current address: Department of Medicine, Division of Gastroenterology, Washington University School of Medicine, Saint Louis, MO 63108, USA

**Keywords:** Crohn's disease, Gut microbiome, Next-generation sequencing, Microbial profiling, 16S rRNA gene

## Abstract

**Background:**

The gut microbiome is altered in Crohn’s disease. Although individual taxa have been correlated with post-operative clinical course, global trends in microbial diversity have not been described in this context.

**Methods:**

We collected mucosal biopsies from the terminal ileum and ascending colon during surgery and post-operative colonoscopy in 6 Crohn’s patients undergoing ileocolic resection (and 40 additional Crohn’s and healthy control patients undergoing either surgery or colonoscopy). Using next-generation sequencing technology, we profiled the gut microbiota in order to identify changes associated with remission or recurrence of inflammation.

**Results:**

We performed 16S ribosomal profiling using 101 base-pair single-end sequencing on the Illumina GAIIx platform with deep coverage, at an average depth of 1.3 million high quality reads per sample. At the time of surgery, Crohn’s patients who would remain in remission were more similar to controls and more species-rich than Crohn’s patients with subsequent recurrence. Patients remaining in remission also exhibited greater stability of the microbiota through time.

**Conclusions:**

These observations permitted an association of gut microbial profiles with probability of recurrence in this limited single-center study. These results suggest that profiling the gut microbiota may be useful in guiding treatment of Crohn’s patients undergoing surgery.

## Background

Crohn’s disease is an inflammatory bowel disease (IBD) with courses of recurrent inflammation, the etiology of which is likely rooted in the interplay of gut microbial imbalances (dysbiosis) and host immunity [1]. Culture-independent studies of the gut microbiota in Crohn’s disease have documented that at least a subset of patients harbor microbial communities distinct from those in individuals without IBD [2-8]. The unpredictability of Crohn’s disease renders sampling of patients prior to the onset of inflammation inherently challenging. Here we addressed this by studying patients undergoing surgery and following them through the initial post-operative colonoscopies. Patients were thereby effectively “synchronized” in the history of their disease by starting at a common event. This design also permitted us to study the onset of inflammation after a state of known health (i.e., following the resection of all diseased bowel). Individual taxa, such as *Faecalibacterium prausnitzii*, have been associated with post-operative clinical course [9]. This finding raises the question that we examined here: can profiling the gut microbiota at large be a useful clinical tool?

We characterized the gut microbiota in 6 patients with Crohn’s disease undergoing surgical resection of the terminal ileum to remove all evident inflammation, a procedure known as ileocolic resection. We collected samples during surgery and the first post-operative colonoscopy. We also characterized 5 Crohn’s patients undergoing surgery alone, 9 patients without IBD undergoing ileocolic resection (due to a colonic polyp or cancer), and 26 patients receiving only colonoscopy (15 with Crohn’s for the purpose of assessing disease activity as per the discretion of the treating gastroenterologist, 11 without IBD receiving colorectal cancer screening) (Additional file 1: Table S1). All patients were recruited via continuous enrollment from a single medical center in the San Francisco Bay Area.

Crohn’s patients undergoing ileocolic resection are at risk for post-operative recurrence of inflammation in the newly terminal ileum: 65% have visible inflammation at colonoscopy after 1 year [10]. Powerful anti-inflammatory treatments can prevent or reduce this but are typically reserved for patients at high risk for recurrence [10,11]. Gastroenterologists use clinical history and colonoscopy (initially performed approximately six months after surgery) to inform treatment decisions. At colonoscopy, mucosal health is graded with the Rutgeerts score (0–1 indicates remission; 2–4, recurrence) [12], which informs prognosis. The aim of the present study is to determine whether profiling the gut microbiota can further inform prognosis in this setting. We asked whether post-operative recurrence could be correlated with microbiota of the resected surgical tissue, either alone or in conjunction with specimens obtained subsequently. Mucosal biopsies were taken from the terminal ileum and ascending colon, distinguishing between inflamed and healthy-appearing mucosa. All biopsies from an individual patient at a given time point were obtained with the same surgical or endoscopic instruments, consistent with previously published protocols [3,5,13-15] but leading to the possibility of cross-contamination. Fecal samples were obtained one day prior to procedures when feasible. We performed microbial profiling of samples based on deep sequencing of a 16S ribosomal gene region. Based on observed associations between species abundance and post-operative recurrence, we propose that microbial profiling of patients with Crohn’s disease undergoing surgery may serve as a prognostic indicator for subsequent clinical course.

## Methods

### Human subjects

Patients scheduled for surgery and/or colonoscopy were recruited to the study from the University of California, San Francisco (UCSF), between November 2008 and August 2010. No patients underwent a procedure specifically for this study. Informed consent was obtained of each patient by one of the authors (ND). The use of human subjects was approved by the Committee on Human Research (CHR) at UCSF (study 10–01519). All samples analyzed were acquired specifically for this study; none were taken from a tissue bank.

### DNA extraction

Stool samples (250 mg) were homogenized in PowerBead tubes (MoBio, Carlsbad, CA) using a Mini-BeadBeater-8 (BioSpec) at the highest setting for 1 minute. DNA was extracted using the MoBio Power Soil DNA Kit (Carlsbad, CA) according to the manufacturer’s instructions. Frozen biopsies (30 mg) were suspended in 600 μl of Buffer RLT (Qiagen, Valencia, CA) that contained β-Mercaptoethanol (1 μl per 100 μl of buffer), and transferred to silica-bead-containing tubes (Lysing Matrix B, MP Biomedicals, Solon, OH). Tubes were placed in a Mini-BeadBeater-8 (BioSpec) and homogenized at the highest setting for 3 minutes. Samples were further thawed in a 37°C water bath for 5 minutes. DNA was subsequently extracted using the column-based Qiagen AllPrep kit (Valencia, CA) as per manufacturer instructions.

### 16S rRNA amplification

A fragment of the 16S ribosomal genes (containing the V1-V3 hypervariable regions) was amplified by PCR with 9F-containing (5′-CAAGCAGAAGACGGCATACGAGATNNNNNNGTGACTGGAGC*GAGTTTGATC [AC]TGGCTCAG-3′*) and 529R-containing (5′-AATGATCGGCGACCACCGAGATCTACACTCTTTC*ACCGCGGC[GT]GCTGGC-3′*) primers at a final concentration of 400 nM using Phusion high-fidelity PCR Master Mix (Finnzymes). (Adapter sequences are underlined. Sample-specific 6-mer barcodes are represented by N’s. 9F and 529R sequences are italicized.) 10 barcodes were used, in varying combinations: AAGCTA, ACATCG, ATTGGC, CACTGT, CGTGAT, CTGATC, GATCTG, GCCTAA, TCAAGT, and TGGTCA. PCR conditions were as follows: 98°C initial denaturation for 30 seconds; 10 cycles of 98°C for 10 seconds, 53°C for 30 seconds, and 72°C for 30 seconds; a final extension at 72°C for 5 minutes. Following PCR, the sample was run on a 1.5% agarose gel (Invitrogen UltraPure agarose, Carlsbad, CA) at 80 Volts for 15 minutes. The amplified fragment was then excised and purified using GeneCatcher tips (the Gel Company, San Francisco, CA). Technical triplicates were performed for each sample.

### Illumina sequencing

Equimolar amounts of amplicons from 6–7 samples were pooled for multiplexing on each flow cell lane. The V3 hypervariable region was sequenced on an Illumina Genome Analyzer IIx using standard reagents. A custom sequencing primer (5′-GATCTACACTCTTTC*ACCGCGGC[GT]GCTGGC*-3′), which contained 529R (italicized), was used to initiate sequencing beyond the 529R primer region, effectively increasing sequencing read length of the region of interest. Despite this strategy, conservation in 16S sequence beyond the 529R primer region required low cluster density on flow cells for successful sequencing. Matrix and phasing values generated from a library control lane were applied to the lanes containing the amplified 16S fragments. We generated 23.3 gigabases of 101 base pair single-end reads of partial 16S ribosomal sequence data.

### 16S rRNA data processing and analysis

Ribosomal sequence data was processed using tools from the QIIME pipeline v1.2.1 [16]. Sequences were de-replicated at 100% sequence identity. No chimeric sequences were seen using ChimeraSlayer [17]. Reads were retained if they were present in at least two samples and represented in at least ten times in the combined dataset. No read trimming or filtering was performed based on Q scores. Reads of presumed human DNA were identified and removed if they both bore 100% sequence identity to human DNA (reference human genome downloaded from http://www.ncbi.nlm.nih.gov/genome/ on May 19, 2011) and were not seen in the Greengenes ribosomal database (downloaded from http://greengenes.lbl.gov/Download/Sequence_Data/Greengenes_format/ on March 17, 2011). Contaminant human DNA constituted 15% of sequences. No contaminant fungal DNA was seen (18S fungal genes downloaded from NCBI on May 19, 2011). 18.4 gigabases of ribosomal sequence data were considered to be high quality and included in our analysis. This total represented a mean coverage of 1.3 million reads per sample ± 0.86 million [s.d.], with a range of 6,400-5,300,000 reads per sample. 14 of the total 139 samples had fewer than 250,000 reads (9 Crohn’s, 5 non-IBD). The mean number of reads per sample was not significantly different in Crohn’s compared to non-IBD controls. The range for samples from the six highlighted Crohn’s patients was 22,000-4,100,000 reads per sample. Reads were aligned using PyNAST [18]. Phylogenetic trees were constructed using FastTree [19]. The unique sequences were assigned to known taxa, to the extent possible, using the RDP classifier [20]. The relative proportions of sequences represented by each taxa were averaged by patient per time point prior to group comparisons, which were made with the Student’s *t*-test (Welch two sample *t*-test) using values from a single time point per patient as indicated in the text.

### Diversity calculations

Alpha diversity was measured using the Chao1 and Faith phylogenetic diversity (PD) metrics [21,22]. Beta diversity was measured using the weighted UniFrac metric [23]. UniFrac distance matrices generated via QIIME were used to compute average distances within and between various groups of samples (e.g. Crohn’s versus control biopsies). The arithmetic mean of coordinates in each dimension of the ordination space (i.e., the PCoA space, a multi-dimensional Euclidean space) was taken to determine the centroid. Autocorrelation, or pseudo-replication, was accounted for by taking the centroid in ordination space of all biopsies collected from a given individual at a given time. For example, in comparing Crohn’s patients to control patients, the centroids of biopsies collected at patients’ initial procedures within this study were used to determine average UniFrac distances. For comparisons that involved multiple pairwise UniFrac distances for an individual, such as inflamed versus healthy-appearing biopsies within a Crohn’s patient, the average was used for the overall calculation. Principal coordinates analysis (PCoA) plots were generated in R [24] using the *ggplot2* package [25]. The Student’s *t*-test with 1,000 Monte Carlo simulations was used to identify statistically significant differences between groups [26,27].

### Heat map generation showing likelihood of recurrence or remission

The two-sided Student’s t-test was used to compare sets of weighted UniFrac distances between surgical biopsies from Crohn’s patients and the following: surgical control biopsies, biopsies from the same patient at the time of the first post-operative colonoscopy, or all control biopsies (i.e., surgery and colonoscopy) (Additional file 2: Figure S4A). In addition, for each Crohn’s patient undergoing surgery, intra-individual comparisons of surgical biopsies were made (Additional file 2: Figure S4B). Likelihood heat maps were generated by overlaying distributions of weighted UniFrac distances in remission and recurrence, then calculating the proportion of UniFrac distances (in bins of width 0.1) seen in remission or recurrence. Leave-one-out cross-validation analyses were performed by generating a predictive model using the aggregate of UniFrac distances from all but one patient, and then “predicting” the clinical outcome of the excluded patient. We generated these predictions by computing the average probability of recurrence arising from each UniFrac distance. Statistical calculations were performed in R [24]. Heat maps were generated using the “heatmap.2” function in the *gplots* package [28].

## Results

### Deep sequencing of a 16S ribosomal RNA gene region

We amplified 16S ribosomal genes with 9F and 529R primers. We used a 529R-containing custom sequencing primer to initiate sequencing beyond the conserved primer sequence region and generate 101 base-pair single-end reads of the V3 hypervariable region using the Illumina GAIIx platform. A total of 139 samples were sequenced from 46 subjects. After filtering, our dataset was comprised of 18.4 gigabases of high-quality reads (mean = 1.3 million reads per sample ± 0.86 million [s.d.]; range 6,400-5,300,000 reads) (see *Methods* for additional details). The combined dataset contained 300,389 unique sequences, or 57,603 operational taxonomic units (OTUs) when clustering by 97% nucleotide sequence identity. This is a higher number of OTUs than previously reported to be associated with human gut [26,29-32], but we suspected this to be a consequence of sequencing error combined with the deep level of coverage [33]. The region of 16S selected evolves at a rate similar to the 16S gene overall [34], so this is not likely to be a major source of difference. We confirmed this by simulating deep sequencing of the gut microbiota. Using previously published datasets of 9,920 [26] and 7,208 [29] near-full-length 16S sequences, we generated large datasets *in silico* of 200 million reads (comparable to our combined dataset of 180 million reads), adding mismatches at rates of 0.01%, 0.1%, or 0.5% per base. Where the original samples contained 451 and 204 OTUs, respectively, the simulated deeply sequenced samples with a 0.1% error rate contained 32,868 and 26,622 OTUs, confirming the prior observation that sequencing noise on the Illumina platform can inflate OTU estimates [35]. With an error rate of 0.5% per base, we observed 554,073 and 431,544 OTUs, respectively, suggesting that – even if the 57,603 OTUs we observed do not represent novel taxa revealed by deep sequencing but instead can largely be accounted for by sequencing error – the true sequencing error rate in our dataset was closer to 0.1% per base. We also performed the inverse experiment, subsampling sets of 10,000 sequences from each of the gut samples we collected, in order to estimate OTU counts that might have been seen with shallower sequencing. These subsamples contained 57–245 (95% confidence interval) OTUs, similar to OTU counts at this sequencing depth seen in prior studies [26,29].

### Inter-individual heterogeneity in Crohn’s disease

**Figure 1:**
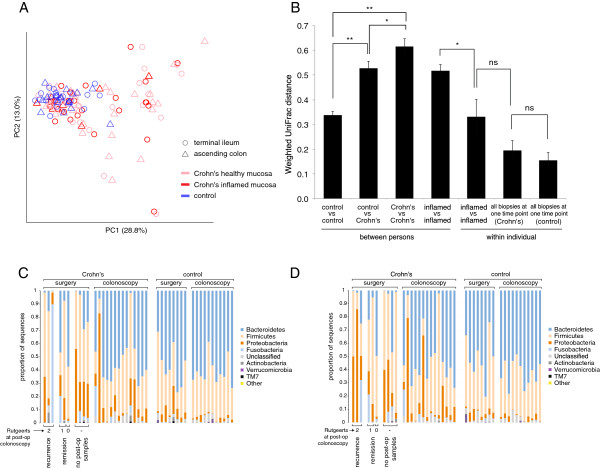
**Inter-individual variability of the gut microbiota is greater in Crohn’s disease than healthy controls. (A)** Principal coordinates analysis (PCoA) plot based on weighted UniFrac distances between biopsies from Crohn’s and non-IBD patients. Biopsies in Crohn’s patients were taken of inflamed and healthy-appearing mucosa. Biopsies did not cluster by inflammation or location. **(B)** Average pairwise weighted UniFrac distances (±s.e.m.) among subsets of biopsies. Asterisks indicate significant differences: * *P*<0.05; ** *P*<0.005 (Student’s *t*-test with 1,000 Monte Carlo simulations). Phylum-level classifications of a single **(C)** ileal or **(D)** colonic sample from each patient’s initial procedure within this study. The Rutgeerts score at post-operative colonoscopy is denoted below surgical samples from Crohn’s patients. Phyla are sorted from top to bottom in overall decreasing prevalence.

We assessed similarity among samples using weighted UniFrac, a beta diversity metric that accounts for both the relative abundances of taxa in each sample and the evolutionary distances among them [23]. In a principal coordinates analysis (PCoA) plot based on pairwise weighted UniFrac distances between samples (Figure 1A), non-IBD patients appeared to cluster together. On the other hand, Crohn’s patients were dispersed throughout the PCoA plot. The gut microbiota in Crohn’s disease does not have a single composition; samples were highly variable, and some were indistinguishable from non-IBD controls. Comparing the centroids in ordination space (i.e., multi-dimensional PCoA space) of all biopsies from each patient’s initial procedure within this study, we found that the average weighted UniFrac distance among Crohn’s patients (“Crohn’s vs Crohn’s” in Figure 1B) was significantly greater than the average distance among control patients (“control vs control”) (*P<*0.005). In fact, the average distance among Crohn’s patients (“Crohn’s vs Crohn’s”) was even greater than the average distance between Crohn’s and control samples (“Crohn’s vs control”) (*P<*0.05), indicating that Crohn’s patients are more different from each other than they are from healthy patients. In particular, Crohn’s patients undergoing surgery seemed to have the greatest variability (Additional file 2: Figure S1). These observations are reflected in Figures 1C and D, which show phylum-level classifications in a single ileal or colonic sample, respectively, from each patient.

Still, several significant differences existed. The two most common phyla in both Crohn’s and control samples were *Bacteroidetes* and *Firmicutes* (Figures 1C-D). All biopsies considered, Crohn’s patients had greater relative abundance of *Proteobacteria* (*P<*10^-4^) and *Fusobacteria* (*P<*0.05), and lower relative abundance of *Bacteroidetes* (*P<*10^-4^), compared to non-IBD patients (Additional file 1: Table S3). Similar to several previous reports [9,36,37], our data showed greater relative abundance of *Faecalibacterium* in healthy patients compared to Crohn’s patients, and Crohn’s remission patients compared to Crohn’s recurrence patients; however, these differences were non-significant. The role of *F. prausnitzii* in the pathogenesis of Crohn’s disease is yet unclear [38]. Consistent with previous observations, the family *Enterobacteriaceae* was more abundant in Crohn’s patients in our study (*P<*10^-3^) [4,7,15]. The genus *Ruminococcus* was less abundant in surgical biopsies from Crohn’s patients relative to non-IBD surgical controls (*P<*0.05), in line with a study reporting lower levels of *Ruminococcus gnavus* in Crohn’s disease [39]. In the context of mixed reports [9,36,40-42], our data showed lower abundance of *Bacteroides* in Crohn’s disease patients (*P<*10^-2^). The presence of discrepancies between studies may represent species- or strain-level differences, or be a consequence of the inter-individual heterogeneity observed here and elsewhere [2,15,43].

Dysbiosis was neither localized nor specific to inflamed mucosa. The average UniFrac distance between inflamed biopsies and adjacent healthy-appearing biopsies from the same individual taken at the same time (“inflamed vs healthy-appearing biopsies” in Additional file 2: Figure S1) was significantly less than the distance between inflamed biopsies from different individuals (“inflamed vs inflamed [between persons]”) (*P<*0.005), indicating that measured microbial community composition is fairly consistent around the ileocolic junction within a patient, regardless of local inflammation. As noted above, this may be due in part to cross-contamination. This held true for both comparisons within the same region (ileum or colon) or between regions (ileum versus colon) (Additional file 2: Figure S1). Prior studies have reported mixed results to this end [5,15,44,45]. These data also suggest that inflamed biopsies in different patients vary in microbial composition, as supported by the comparison of the average distance between inflamed biopsies from different individuals (“inflamed vs inflamed [between persons]” in Figure 1B) with the average distance between inflamed biopsies from an individual taken at a given time point (“inflamed vs inflamed [within individual]”) (*P<*0.05). Indeed, inflamed biopsies from different patients did not cluster together, but rather varied across PCoA space (Figure 1A), suggesting multiple varieties of dysbiosis. Finally, stool and biopsies acquired concomitantly from a single individual were no more similar to each other than biopsies (or stool) from different individuals (Additional file 2: Figures S1, S2).

### Surgical biopsies from Crohn’s patients who remain in remission are more similar to controls

**Figure 2:**
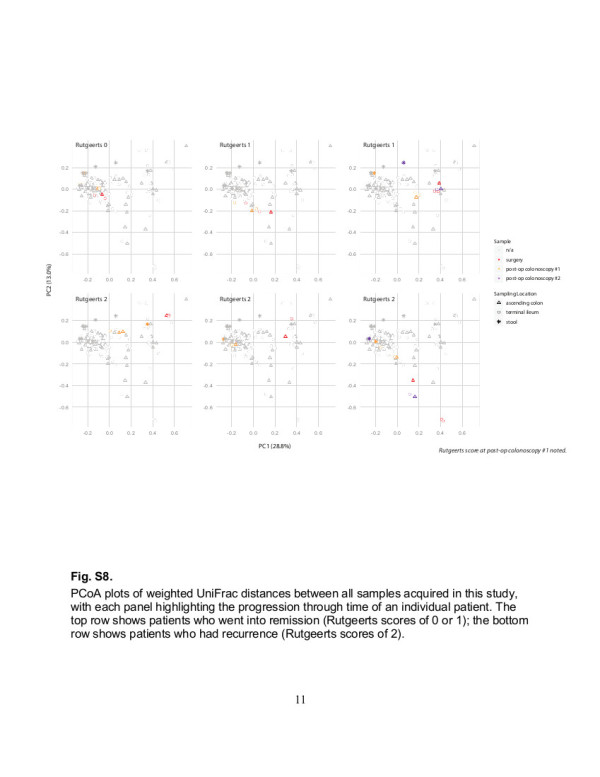
**Crohn’s patients with recurrence have significant dysbiosis at the time of surgery relative to control patients. (A)** PCoA plot of weighted UniFrac distances between all surgical biopsies and non-IBD colonoscopic biopsies, with Crohn’s surgical samples (red) sized according to Rutgeerts score at post-operative colonoscopy. A Rutgeerts score of 2 or more indicates recurrence; less than 2, remission. **(B)** Average weighted UniFrac distances (±s.e.m.) between Crohn’s surgical samples and surgical controls were significantly greater in recurrence. The asterisk indicates a significant difference: * *P*<0.05. **(C)** Heat map showing probability of remission or recurrence based on weighted UniFrac distance to a single surgical control biopsy, binned in increments of 0.1. Each row adds up to 1. Based on a single biopsy per Crohn’s patient, differences in UniFrac distance distributions between remission and recurrence were not consistently significant, as demonstrated by **(D)** random selection of a single surgical biopsy from each of the six Crohn’s patients and determination of UniFrac distance to a randomly selected control biopsy (repeated 10,000 times), and **(E)** examination of all 486 combinations of pairwise comparisons between each surgical biopsy from the six Crohn’s patients and the centroid in ordination space of control biopsies. Dark gray shaded bars indicate a *P*-value of less than 0.05.

Of the 11 Crohn’s patients who underwent surgery, 6 had follow-up at our center during the study period and provided usable samples (Additional file 1: Table S2; Additional file 2: Figure S3 describes the other patients). All six patients were on biological therapies at the time of surgery: 5 were on adalimumab (anti-TNF-α) prior to surgery; 1 had previously been on adalimumab but had recently switched to natalizumab (anti-α4-integrin). Half of these patients experienced recurrence at post-operative colonoscopy. Surgical biopsies from patients with recurrence appeared to be outliers on the PCoA plot relative to the main cluster of samples constituted largely by non-IBD samples (Figure 2A). The average UniFrac distance between surgical samples from Crohn’s patients and from non-IBD controls was significantly greater in recurrence than in remission (*P*=0.03; Figures 2B, Additional file 2: Figure S4A). Patients who subsequently enjoyed remission at 6 months were more similar to the controls. Large UniFrac distances to controls were rarely seen in remission, while small distances were rare in recurrence (Figure 2C). Nonetheless, there was an overlapping distribution of UniFrac distances to controls in remission and recurrence.

Given these patterns, we asked whether a single surgical biopsy from a Crohn’s patient could be used to estimate probability of recurrence or remission. We took two approaches to studying this. First, we randomly selected a single surgical biopsy from each of the six Crohn’s patients and determined the UniFrac distance to a randomly selected control biopsy. We then compared the distributions of three distances from remission patients versus three distances from recurrence patients. A different control biopsy was used for each comparison, in order to test whether any observed differences were robust to variation among controls. Repeating this exercise 10,000 times, we found that these distributions were significantly different in 34% of cases, as defined by a *P* value of less than 0.05 (Figure 2D). Second, we determined UniFrac distances from each Crohn’s surgical biopsy to the centroid in ordination space of all control biopsies. We compared distributions in remission versus recurrence, examining all 486 combinations of pairwise comparisons between each surgical sample from the six Crohn’s patients and the centroid of control biopsies (Figure 2E). The distributions of distances in remission and recurrence were significantly different in 56% of comparisons. Both of these calculations suggest that use of a single biopsy specimen for prognostic purposes may not be reliably useful.

We therefore examined the utility of combining information from multiple biopsies. For each of the six Crohn’s patients, we estimated outcome based on likelihood models constructed from the other five patients (Additional file 2: Figures S5, S6). In this leave-one-patient-out cross-validation analysis, pairwise UniFrac distances between surgical biopsies of the five-patient “training set” and all controls were used to generate heat maps reflecting probabilities of recurrence given a single UniFrac distance. We then computed the probability of recurrence by averaging the probabilities generated by each surgical biopsy from the “test patient.” This scheme correctly predicted recurrence or remission in all cases. In two cases, the correct prediction was made with low confidence, based on a probability of 53-55%; in the remaining cases, the signal was substantially stronger, such that the mean probability associated with all predictions in this leave-one-patient-out analysis was 79% ± 9%. Multiple biopsies appear to improve signal despite the general trend that biopsies from a patient are most similar to each other. This may relate to the inconsistency seen between biopsies from surgery in patients with recurrence, discussed below.

### Crohn’s patients with post-operative recurrence have less consistency between surgical biopsies and less stability through time

**Figure 3:**
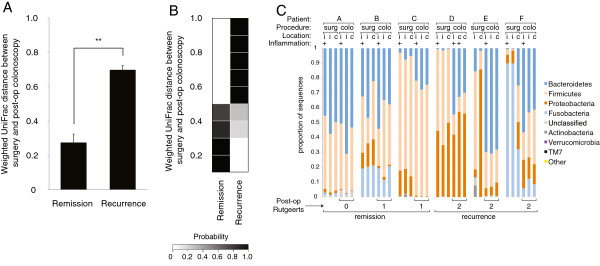
**Recurrence is associated with instability through time. (A)** Average weighted UniFrac distances (±s.e.m.) between surgical and post-operative colonoscopy biopsies for an individual. The asterisks indicate a significant difference: ** *P*<0.005. **(B)** Heat map showing probability of remission or recurrence based on a single pairwise weighted UniFrac distance at surgery and post-operative colonoscopy, binned in increments of 0.1. Each row adds up to 1. **(C)** Phylum-level classifications of biopsies acquired at surgery (“surg”) and post-operative colonoscopy (“colo”) suggest greater stability in remission. Location (i = ileum; c = colon) and presence of inflammation at the biopsy site are indicated.

Crohn’s patients with recurrence exhibited less stable microbiota through time. The average UniFrac distance between the centroid of a patient’s surgical biopsies and the centroid of the same patient’s post-operative biopsies was greater in recurrence than in remission (*P<*0.005; Figure 3A). A similar but non-significant trend was seen when we averaged the pairwise UniFrac distances among surgical samples from an individual (i.e., the average of all pairwise distances between inflamed ileum, healthy ileum, and healthy colon from a single patient at the time of surgery): Crohn’s patients with recurrence showed less consistency (Additional file 2: Figure S4B). This may underlie the above finding that multiple biopsies improve prediction strength. These observations are reflected in the taxonomic classifications of biopsies acquired from patients included in the longitudinal analysis: patients who stay in remission appear to have a more even profile across samples (Figures 1C-D, 3C). We used the distribution of UniFrac distances through time (i.e., surgery compared to post-operative colonoscopy for an individual) to generate heat maps showing probability of clinical outcomes (Figure 3B, Additional file 2: Figure S4B). We validated our model using a leave-one-patient-out approach as above. Again, predictions were correct with varying levels of confidence. In one case, confidence was low at 59%; in the remainder, the signal was substantially stronger, such that the mean probability associated with all predictions was 88% ± 8% (Additional file 2: Figure S7). Instability of the gut microbiota through time may therefore be related to recurrence. Interestingly, biopsies from post-operative colonoscopies of all Crohn’s patients reflected a migration toward the cluster of control biopsies, regardless of Rutgeerts score (Additional file 2: Figure S8).

### Reduced species richness in Crohn’s pronounced in recurrence

Consistent with prior reports [2,4,15,46], we observed reduced alpha diversity (i.e., species richness) in the gut microbiota of Crohn’s patients. In light of variations in sequencing depth, we subsampled sets of 10,000 sequences from each sample, excluding the single sample with fewer total reads (a colonic biopsy from a patient without IBD). We observed 143 +/− 5 (s.e.m.) OTUs in patients with Crohn’s disease, as compared to 176 +/− 6 (s.e.m.) OTUs in non-IBD controls. The Chao1 nonparametric estimator and the Faith phylogenetic diversity (PD) metric also indicated reduced diversity in Crohn's patients compared to controls (Additional file 2: Figures S9, S10) [21,22]. The reduction in alpha diversity in Crohn’s was significant in patients with recurrence with both Chao1 and PD metrics relative to non-IBD controls (*P*<0.05). These data are consistent with the hypothesis that a rich gut microbiota may protect against disease.

We identified a limited number of significant taxonomic differences in samples from Crohn’s patients with recurrence relative to those who stayed in remission. At surgery, patients who would go on to experience recurrence had a significantly lower relative abundance of the families *Lachnospiraceae* (*P<*10^-2^) and *Erysipelotrichaceae* (*P<*0.05), as well as an unidentified genus within *Clostridia* (*P<*10^-2^), all members of the phylum *Firmicutes* (Additional file 1: Table S3). Recurrence was associated with a greater abundance of *Rhodobacteraceae* (of class *Alphaproteobacteria*; *P*<0.05) and an unknown *Proteobacteria* (*P*<10^-2^). At post-operative colonoscopy, the genus *Rhizobium* (also *Alphaproteobacteria*) was the lone significant difference between Crohn’s patients experiencing recurrence and those remaining in remission, more abundant in patients with recurrence (*P*<0.05).

## Discussion

Understanding the gut microbiome in IBD is of interest to both clinicians and patients, who are eager to predict clinical course of the disease and identify appropriate treatments to improve health outcomes, including probiotics and diet [47]. Studying the etiologic role of the gut microbiome in Crohn’s disease is challenging, due to multiple issues including unpredictability of disease course and patient selection. Many studies have characterized patients at a single time point and attributed to Crohn’s disease the observed microbial differences from controls [2,4,7,15,36,37,42-46]. Global trends in diversity have been underexplored. Here, we followed carefully phenotyped patients over time to correlate microbial profiles with clinical course, synchronizing the time course with ileocolic resection, and controlling for biologic medication usage and geographic impact. Consistent with prior reports suggesting translational utility of gut microbial profiling in Crohn’s disease [9,48], we show here that patients experiencing recurrence of inflammation after surgical resection of all diseased bowel have distinct diversity profiles relative to patients who remain in remission. A clinical test characterizing multiple biopsies from a surgically resected specimen may ultimately be useful for informing prognosis.

While this study addresses several challenges of study design pertaining to variability and unpredictability in disease course, it is also subject to certain limitations. The findings presented here represent a small number of patients on biologic therapies at a single medical center. Conceivably, these findings may therefore only apply to a limited subset of Crohn’s patients, whether it be patients on related medications or those in the study’s catchment population. Given differences in study design, patient characteristics, sequencing strategies, and other experimental variables, it is difficult to directly evaluate patients described in previous studies in the context of our findings. The development of a practical test based on these findings will require validation on a larger and more diverse patient population involving multiple medical centers.

There were several questions we were unable to address. We wished to collect fecal and biopsy samples at all time points in order to examine the relationships of both to outcomes. Unfortunately, we were only able collect fecal samples from already-enrolled Crohn’s patients concurrent with their post-operative colonoscopy. Subsequent investigations examining the predictive capacity of stool may build upon current knowledge of fecal biomarkers [49], perhaps by focusing on patients with inflammation of the distal gut or in patients who are not otherwise scheduled for an invasive evaluation (e.g. colonoscopy).

We also could not interpret the observed post-operative “migration” of samples on the PCoA plot towards the healthy controls in cases of both recurrence and remission. It is unclear whether this constituted some degree of recovery from dysbiosis. Pre-operative data on the Crohn’s patients or longitudinal data on the non-IBD surgical controls may have enabled a better understanding of this migration. Future studies should evaluate pharmacologic or dietary interventions in the context of microbial shifts through time. Recent reviews of probiotics and prebiotics in Crohn’s disease have underscored a lack of knowledge in this area [50-53].

## Conclusions

In summary, we demonstrated in this limited cohort at a single medical center that analyses of surgical biopsies using the UniFrac metric are associated with subsequent clinical course. Whether the severity of dysbiosis or the instability of the gut microbiota (or both) is the critical determinant of recurrence in Crohn’s patients undergoing surgery will need to be further explored. Whether these influence inflammation or are the result thereof remains unclear. Dysbiosis in Crohn’s has many varieties and does not appear to be specific to inflamed mucosa. With apologies to Tolstoy, “unhappy guts” in Crohn’s appear to be “unhappy” in different ways [54]. Taken together with the broad spectrum of genetic mutations associated with Crohn’s disease [55,56], it is not surprising that a specific cause has not been identified, and that IBD patients require individualized care. Further studies are warranted to validate these conclusions in a larger set of patients and other clinical contexts.

## Abbreviations

IBD: Inflammatory bowel disease; OTU: Operational taxonomic unit; PCoA: Principal coordinates analysis.

## Competing interests

The authors declare that they have no competing interests.

## Authors’ contributions

ND was involved in study concept and design; patient consent; sample collection and processing; acquisition, analysis, and interpretation of data; and drafting and revising the manuscript. DAWS and SR were involved in analysis and interpretation of data, and revision of the manuscript. SEB was involved in study concept and design; analysis and interpretation of data; drafting and revising the manuscript; and study supervision. All authors contributed to and approved the final manuscript by providing constructive suggestions.

## Pre-publication history

The pre-publication history for this paper can be accessed here:

http://www.biomedcentral.com/1471-230X/13/131/prepub

## Supplementary Material

Additional file 1: Table S1 Demographics and clinical characteristics of study patients (numbers in parentheses), with a focus on the two sets of patients discussed most extensively in the text: **(A)** Crohn’s patients with recurrence or remission, and **(B)** Crohn’s patients and non-IBD control patients. **Table S2**. Demographic and clinical data on patients studied longitudinally. **Table S3**. P-values of differences in relative abundances of taxa between **(A)** surgical biopsies from Crohn’s patients with recurrence and those in remission (time point 1), **(B)** colonoscopic biopsies from Crohn’s patients with recurrence and those in remission (time point 2), **(C)** surgical biopsies from Crohn’s patients and controls, or **(D)** all biopsies from Crohn’s patients and controls. Taxonomic strings are listed to the deepest taxonomic rank that could be determined. Significant differences (P < 0.05) are indicated in this table and color-coded to indicate which group contains the higher proportion of the microbe: red for recurrence **(A,B)** or Crohn’s **(C,D)**; blue for remission **(A,B)** or controls **(C,D)**; and gray for non-significant. Taxa that did not have significant differences in any of the four categories are not included in the table. Click here for file

Additional file 2: Figure S1 Average weighted UniFrac distances (±s.e.m.) among subsets of biopsies. Asterisks indicate significant differences: * P<0.05; ** P<0.005 (Student’s t-test with 1,000 Monte Carlo simulations). **Figure S2**. PCoA plot of weighted UniFrac distances **(A)** between all samples acquired in this study (stool is indicated with an asterisk) and **(B)** between stool samples and biopsies acquired at the same time in 5 patients with Crohn’s disease, at the first 1–2 postoperative colonoscopies. **Figure S3**. PCoA plot of weighted UniFrac distances between all biopsies (i.e., from all patients at all time points) denoting samples from Crohn’s patients who underwent surgery but were excluded from predictive modeling. 1 patient was lost to follow-up. The remaining 4 underwent post-op colonoscopy and were given Rutgeerts scores of 0, 1, 2, and 3, referred to as patients 0–3, respectively, in this discussion. Patient 0 was scheduled for ileocolic resection when recruited to this study. However, an intra-operative decision was made to perform a limited ileal resection combined with stricturoplasty. During postop colonoscopy, the scope could not be advanced to the surgical anastomosis, and so the Rutgeerts score (which was based on the visualized terminal ileum) was not reflective of mucosal health at the anastomosis. Patient 1 agreed to provide surgical specimens but declined to provide biopsies at post-op colonoscopy. Patient 2 had postop colonoscopy after the sample collection period had ended. Patient 3 received postop colonoscopy at another hospital but was described to have significant inflammation and narrowing at the anastomosis, consistent with a score of 3. **Figure S4**. Average weighted UniFrac distances (±s.e.m.) **(A)** between Crohn’s surgical and all control biopsies, and **(B)** among surgical biopsies from an individual Crohn’s patient, demonstrating differences in remission versus recurrence. The asterisk indicates a significant difference: * P<0.05; ns, non-significant. **Figure S5**. Leave-one-out validation testing of heat maps for generating predictions regarding postoperative recurrence in Crohn’s patients using weighted UniFrac distances between biopsies taken at surgery and surgical controls. The post-operative Rutgeerts score is shown on the left (remission = 1–0; recurrence = 2 or more). The distribution of UniFrac distances for each patient is represented as the top box plot in each set of three. The subsequent two box plots in each set are the distributions of UniFrac distances in the remaining 5 patients, distinguishing those who had recurrence from those in remission. Heat maps on the right were generated from these distributions to reflect probabilities of recurrence or remission given a single UniFrac distance. **Figure S6**. Leave-one-out validation testing of heat maps for generating predictions regarding postoperative recurrence in Crohn’s patients using weighted UniFrac distances between biopsies taken at surgery and all control biopsies (taken from surgery and colonoscopy). The post-operative Rutgeerts score is shown on the left (remission = 0–1; remission = 2 or more). The distribution of UniFrac distances for each patient is represented as the top box plot in each set of three. The subsequent two box plots in each set are the distributions of UniFrac distances in the remaining 5 patients, distinguishing those who had recurrence from those in remission. Heat maps on the right were generated from these distributions to reflect probabilities of recurrence or remission given a single UniFrac distance. **Figure S7**. Leave-one-out validation testing of heat maps for generating predictions regarding postoperative recurrence in Crohn’s patients using weighted UniFrac distances between biopsies taken at surgery and at post-operative colonoscopy. The post-operative Rutgeerts score is shown on the left (remission = 0–1; remission = 2 or more). The distribution of UniFrac distances for each patient is represented as the top box plot in each set of three. The subsequent two box plots in each set are the distributions of UniFrac distances in the remaining 5 patients, distinguishing those who had recurrence from those in remission. Heat maps on the right were generated from these distributions to reflect probabilities of recurrence or remission given a single UniFrac distance. **Figure S8**. PCoA plots of weighted UniFrac distances between all samples acquired in this study, with each panel highlighting the progression through time of an individual patient. The top row shows patients who went into remission (Rutgeerts scores of 0 or 1); the bottom row shows patients who had recurrence (Rutgeerts scores of 2). **Figure S9**. Alpha diversity as measured using the chao1 metric, comparing **(A)** control versus Crohn’s samples and **(B)** surgical biopsies of Crohn’s patients who go on to have recurrence versus those who stay in remission, compared to surgical control biopsies. The asterisk indicates a significant difference: * P<0.05. **Figure S10**. Alpha diversity as measured using phylogenetic diversity, comparing **(A)** control versus Crohn’s samples and **(B)** surgical biopsies of Crohn’s patients who go on to have recurrence versus those who stay in remission, compared to surgical control biopsies. The asterisk indicates a significant difference: * P<0.05. Click here for file

## Data deposition

We have obtained a letter of certification to submit the combined 16S rRNA sequence dataset to the NCBI database of Genotypes and Phenotypes (dbGaP). Data are being submitted at the time of publication.
