# Supplementary material for: Association of gut microbiota with post-operative clinical course in Crohn’s disease
Source: BMC Gastroenterol. 2013 Aug 22;13:131. doi: 10.1186/1471-230X-13-131 (PMC3848607; doi:10.1186/1471-230X-13-131)
Supplement: Additional file 1: Table S1 — Demographics and clinical characteristics of study patients (numbers in parentheses), with a focus on the two sets of patients discussed most extensively in the text: (A) Crohn’s patients with recurrence or remission, and (B) Crohn’s patients and non-IBD control patients. Table S2. Demographic and clinical data on patients studied longitudinally. Table S3. P-values of differences in relative abundances of taxa between (A) surgical biopsies from Crohn’s patients with recurrence and those in remission (time point 1), (B) colonoscopic biopsies from Crohn’s patients with recurrence and those in remission (time point 2), (C) surgical biopsies from Crohn’s patients and controls, or (D) all biopsies from Crohn’s patients and controls. Taxonomic strings are listed to the deepest taxonomic rank that could be determined. Significant differences (P < 0.05) are indicated in this table and color-coded to indicate which group contains the higher proportion of the microbe: red for recurrence (A,B) or Crohn’s (C,D); blue for remission (A,B) or controls (C,D); and gray for non-significant. Taxa that did not have significant differences in any of the four categories are not included in the table. [file 1471-230X-13-131-S1.pdf]

Table S1.

Demographics and clinical characteristics of study patients (numbers in parentheses), with a focus on the two sets of patients discussed most extensively in the text: (*A*) Crohn's patients with recurrence or remission, and (*B*) Crohn's patients and non-IBD control patients.

(A)

	Recurrence (3)	Remission (3)	p-value
Male sex (%)	100	33	0.18
White race (%)	100	67	0.42
Mean age	29	37	0.47
Median age	29	29	n/a
Mean latitude of birth city	39	39	0.77
Median latitude of birth city	38	40	n/a
International travel (%)	0	33	0.42
Mean disease duration	12	20	0.63
Median disease duration	16	14	n/a
Harvey-Bradshaw Index	10	5	0.18
5-ASA (%)	33	0	0.42
Methotrexate (%)	0	0	n/a
Immunomodulator (%)	0	0	n/a
anti-TNF-α biologic (%)	100	67	0.42
anti-α4-integrin biologic (%)	0	33	0.42
Steroids (%)	0	0	n/a
Recent antibiotics (%)	67	67	1.00
Recent probiotics (%)	100	67	0.42
History of intestinal surgery (%)	33	33	1.00

(B)

	Crohn's (26)	Control (20)	p-value
Male sex (%)	50.0	20.0	0.03
White race (%)	69.2	65.0	0.78
Mean age	34.6	55.2	4.59×10^{-6}
Median age	29.5	53.0	n/a
Mean latitude of birth city	38.2	37.0	0.66
Median latitude of birth city	38.0	38.0	n/a
International travel (%)	38.5	40.0	0.92
Mean disease duration	9.3	0.0	n/a
Median disease duration	6.0	0.0	n/a
Harvey-Bradshaw Index	5.3	0.8	4.79×10^{-6}
5-ASA (%)	34.6	0.0	1.25×10^{-3}
Methotrexate (%)	3.8	0.0	0.33
Immunomodulator (%)	15.4	0.0	0.04
anti-TNF-α biologic (%)	53.8	0.0	1.33×10^{-5}
anti-α4-integrin biologic (%)	3.8	0.0	0.33
Steroids (%)	15.4	0.0	0.04
Recent antibiotics (%)	65.4	25.0	0.01
Recent probiotics (%)	61.5	45.0	0.28
History of intestinal surgery (%)	42.3	5.0	1.80×10^{-3}

Table S2.
Demographic and clinical data on patients studied longitudinally.

Study subject ID	201	635	672	667	611	615
Post-operative recurrence or remission	remission	remission	remission	recurrence	recurrence	recurrence
Type (e.g. structuring)	stricturing	stricturing and penetrating	stricturing	stricturing	penetrating	stricturing
Montreal classification, pre-operative *	L1	L1	L3	L1	L3	L1
Duration of disease (years)	14	2	44	18	16	3
History of IBD medications previously used	prednisone, 6MP, adalimumab, infliximab	prednisone, 6MP, infliximab	mesalamine, prednisone, budesonide, 6MP, infliximab	mesalamine, prednisone, budesonide, 6MP, infliximab	mesalamine, prednisone, 6MP, infliximab	budesonide, prednisone, 6MP
Pre-operative medical IBD therapy	natalizumab	adalimumab	adalimumab	adalimumab; mesalamine	adalimumab	adalimumab
Post-operative medical IBD therapy	certolizumab	adalimumab	adalimumab	none	adalimumab	adalimumab
Smoking history	none	none	none	none	none	none
Antibiotics within 3 months prior to surgery	ciprofloxacin, metronidazole	none	ciprofloxacin	ciprofloxacin, metronidazole	ciprofloxacin	none
Probiotic capsules or yogurt within 3 months prior to surgery	capsules	capsules	none	capsules	capsules and yogurt	yogurt
History of intestinal surgery	none	none	ileal resection	ileal resection	none	none
Indication(s) for surgery	obstruction	obstruction; fistula; failure of medical management	obstruction	obstruction	failure of medical management	obstruction
Harvey-Bradshaw Index score	4	7	4	14	12	5
Histology of surgical specimen	chronic active ileitis, acute ulceration, polypoid granulation tissue, transmural lymphoid aggregates, and non-necrotizing granulomas	focal chronic and active ileitis with transmural inflammation; ileocolic fistula	fibrostenotic stricture in terminal ileum; otherwise normal	chronic and active colitis; fibrostenotic stricture in terminal ileum	ulcer, fissure, fistula, noncaseating granulomas in ileum and cecum	chronic and active colitis; fibrostenotic stricture in terminal ileum
Timing of first post-operative colonoscopy (months after surgery)	6	5	6	10	6	7
Rutgeerts score	1	0.5	1	2	2	2
Histology from first post-operative colonoscopy	normal ileum and colon	normal ileum and colon	minimal inflammation at anastomosis	active ileitis; active colitis with non-necrotizing granulomas	mild active ileitis	mild active ileitis

* Note that histologic reports of surgically resected specimens sometimes describe microscopic colitis despite a pre-operative L1 classification

L1=ileal; L2=colonic; L3=ileocolonic; L4=isolated upper GI disease

Table S3.

P-values of differences in relative abundances of taxa between (*A*) surgical biopsies from Crohn's patients with recurrence and those in remission (time point 1), (*B*) colonoscopic biopsies from Crohn's patients with recurrence and those in remission (time point 2), (*C*) surgical biopsies from Crohn's patients and controls, or (*D*) all biopsies from Crohn's patients and controls. Taxonomic strings are listed to the deepest taxonomic rank that could be determined. Significant differences ($P < 0.05$) are indicated in this table and color-coded to indicate which group contains the higher proportion of the microbe: red for recurrence (*A*,*B*) or Crohn's (*C*,*D*); blue for remission (*A*,*B*) or controls (*C*,*D*); and gray for non-significant. Taxa that did not have significant differences in any of the four categories are not included in the table.

	Taxon	(A) Crohn's: recurrence vs remission<br>surgical biopsies (i.e., time point 1)	(B) Crohn's: recurrence vs remission<br>colo biopsies (i.e., time point 2)	(C) Crohn's vs controls<br>surgical biopsies	(D) Crohn's vs controls<br>all biopsies
Phylum	Root_Bacteria_Bacteroidetes	2.10E-01	5.52E-01	5.50E-05	3.04E-05
	Root_Bacteria_Fusobacteria	5.62E-01	6.65E-01	1.74E-01	2.56E-02
	Root_Bacteria_Proteobacteria	1.72E-01	2.24E-01	4.66E-03	8.81E-05
	Root_Bacteria_Unclassified	6.43E-01	4.54E-01	3.10E-02	4.58E-02
Class	Root_Bacteria_Bacteroidetes	2.11E-01	4.73E-01	3.54E-05	3.36E-05
	Root_Bacteria_Bacteroidetes_Flavobacteria	5.57E-01	6.02E-01	3.89E-02	3.40E-02
	Root_Bacteria_Firmicutes_Erysipelotrichi	4.09E-02	8.33E-01	7.85E-01	5.91E-01
	Root_Bacteria_Fusobacteria	5.62E-01	6.65E-01	1.74E-01	2.56E-02
	Root_Bacteria_Proteobacteria_Deltaproteobacteria	4.91E-01	6.43E-01	8.06E-02	2.86E-02
	Root_Bacteria_Proteobacteria_Gammaproteobacteria	1.60E-01	2.21E-01	5.67E-03	9.12E-05
	Root_Bacteria_Proteobacteria_Unclassified	9.00E-03	1.95E-01	2.96E-01	1.06E-02
	Root_Bacteria_Unclassified	6.43E-01	4.54E-01	3.10E-02	4.58E-02
Order	Root_Bacteria_Bacteroidetes_Bacteroidetes_Bacteroidales	2.11E-01	4.73E-01	3.54E-05	3.36E-05
	Root_Bacteria_Bacteroidetes_Flavobacteria_Flavobacteriales	5.57E-01	6.02E-01	3.89E-02	3.40E-02
	Root_Bacteria_Firmicutes_Clostridia_Unclassified	8.48E-03	2.13E-01	6.48E-01	1.44E-01
	Root_Bacteria_Firmicutes_Erysipelotrichi_Erysipelotrichales	4.09E-02	8.33E-01	7.85E-01	5.91E-01
	Root_Bacteria_Fusobacteria_Fusobacteria_Fusobacteriales	5.62E-01	6.65E-01	1.74E-01	2.56E-02
	Root_Bacteria_Proteobacteria_Alphaproteobacteria_Rhodobacterales	3.37E-02	9.14E-01	3.17E-01	3.26E-01
	Root_Bacteria_Proteobacteria_Alphaproteobacteria_Sphingomonadales	7.45E-01	4.57E-01	7.62E-02	4.22E-02
	Root_Bacteria_Proteobacteria_Deltaproteobacteria_Desulfovibrionales	5.43E-01	5.69E-01	8.16E-02	2.78E-02
	Root_Bacteria_Proteobacteria_Gammaproteobacteria_Enterobacteriales	1.66E-01	2.25E-01	8.03E-03	1.38E-04
	Root_Bacteria_Proteobacteria_Gammaproteobacteria_Unclassified	1.27E-01	1.39E-01	1.38E-02	6.57E-05
	Root_Bacteria_Proteobacteria_Unclassified	9.00E-03	1.95E-01	2.96E-01	1.06E-02
	Root_Bacteria_Unclassified	6.43E-01	4.54E-01	3.10E-02	4.58E-02
Family	Root_Bacteria_Bacteroidetes_Bacteroidetes_Bacteroidales_Bacteroidaceae	2.49E-01	4.27E-01	2.56E-03	3.85E-03
	Root_Bacteria_Bacteroidetes_Flavobacteria_Flavobacteriales_Flavobacteriaceae	5.57E-01	6.00E-01	3.88E-02	3.38E-02
	Root_Bacteria_Firmicutes_Bacilli_Lactobacillales_Enterococcaceae	4.25E-01	3.54E-01	1.69E-01	2.52E-02
	Root_Bacteria_Firmicutes_Clostridia_Clostridiales_Lachnospiraceae	7.93E-03	1.22E-01	2.52E-01	2.58E-01
	Root_Bacteria_Firmicutes_Clostridia_Unclassified	8.48E-03	2.13E-01	6.48E-01	1.44E-01
	Root_Bacteria_Firmicutes_Erysipelotrichi_Erysipelotrichales_Erysipelotrichaceae	4.09E-02	8.33E-01	7.85E-01	5.91E-01
	Root_Bacteria_Fusobacteria_Fusobacteria_Fusobacteriales_Fusobacteriaceae	3.56E-01	7.02E-01	8.86E-02	1.08E-02
	Root_Bacteria_Proteobacteria_Alphaproteobacteria_Rhizobiales_Rhizobiaceae	9.03E-01	1.76E-02	9.60E-02	6.82E-02
	Root_Bacteria_Proteobacteria_Alphaproteobacteria_Rhodobacterales_Rhodobacteraceae	3.37E-02	9.14E-01	3.17E-01	3.26E-01
	Root_Bacteria_Proteobacteria_Alphaproteobacteria_Sphingomonadales_Sphingomonadaceae	7.45E-01	4.57E-01	7.62E-02	4.22E-02
	Root_Bacteria_Proteobacteria_Deltaproteobacteria_Desulfovibrionales_Desulfovibrionaceae	5.43E-01	5.62E-01	8.32E-02	2.76E-02
	Root_Bacteria_Proteobacteria_Gammaproteobacteria_Enterobacteriales_Enterobacteriaceae	1.66E-01	2.25E-01	8.03E-03	1.38E-04
	Root_Bacteria_Proteobacteria_Gammaproteobacteria_Unclassified	1.27E-01	1.39E-01	1.38E-02	6.57E-05
	Root_Bacteria_Proteobacteria_Unclassified	9.00E-03	1.95E-01	2.96E-01	1.06E-02
	Root_Bacteria_Unclassified	6.43E-01	4.54E-01	3.10E-02	4.58E-02
Genus	Root_Bacteria_Acidobacteria_Acidobacteria_Acidobacteriales_Acidobacteriaceae_Unclassified	8.14E-01	6.43E-01	1.42E-01	4.11E-02
	Root_Bacteria_Actinobacteria_Actinobacteria_Actinobacteridae_Actinomycetales_Corynebacterineae	8.63E-01	2.57E-01	6.63E-02	4.27E-02
	Root_Bacteria_Bacteroidetes_Bacteroidetes_Bacteroidales_Bacteroidaceae_Bacteroides	2.49E-01	4.27E-01	2.56E-03	3.85E-03
	Root_Bacteria_Bacteroidetes_Bacteroidetes_Bacteroidales_Bacteroidaceae_Unclassified	5.00E-01	6.24E-01	2.34E-02	1.53E-01
	Root_Bacteria_Firmicutes_Bacilli_Lactobacillales_Enterococcaceae_Enterococcus	4.25E-01	3.53E-01	1.69E-01	2.54E-02
	Root_Bacteria_Firmicutes_Bacilli_Lactobacillales_Enterococcaceae_Unclassified	4.23E-01	3.70E-01	2.01E-01	2.83E-02
	Root_Bacteria_Firmicutes_Clostridia_Clostridiales_Lachnospira	5.93E-01	4.31E-01	5.46E-02	3.45E-02
	Root_Bacteria_Firmicutes_Clostridia_Clostridiales_Lachnospiraceae_Lachnospira_Unclassified	5.93E-01	4.31E-01	5.46E-02	3.45E-02
	Root_Bacteria_Firmicutes_Clostridia_Clostridiales_Lachnospiraceae_Unclassified	1.37E-02	6.45E-02	3.09E-02	1.78E-01
	Root_Bacteria_Firmicutes_Clostridia_Clostridiales_Ruminococcaceae_Ruminococcus	9.00E-01	4.23E-01	2.54E-02	6.69E-01
	Root_Bacteria_Firmicutes_Clostridia_Clostridiales_Ruminococcaceae_Subdoligranulum	4.46E-01	4.25E-01	1.27E-02	1.50E-02
	Root_Bacteria_Firmicutes_Clostridia_Clostridiales_Veillonellaceae_Dialister	2.92E-01	9.07E-01	9.15E-01	3.38E-02
	Root_Bacteria_Firmicutes_Clostridia_Unclassified	8.48E-03	2.13E-01	6.48E-01	1.44E-01
	Root_Bacteria_Firmicutes_Erysipelotrichi_Erysipelotrichales_Erysipelotrichaceae_ErysipelotrichaceaeIncertaeSedis	4.64E-02	9.48E-01	9.53E-01	9.19E-01
	Root_Bacteria_Firmicutes_Erysipelotrichi_Erysipelotrichales_Erysipelotrichaceae_Holdemania	9.15E-01	4.24E-01	1.72E-01	3.31E-02
	Root_Bacteria_Fusobacteria_Fusobacteria_Fusobacteriales_Fusobacteriaceae_Fusobacterium	3.55E-01	7.47E-01	9.90E-02	1.18E-02
	Root_Bacteria_Proteobacteria_Alphaproteobacteria_Rhizobiales_Hyphomicrobiaceae_Unclassified	8.51E-01	4.23E-01	6.15E-02	3.36E-02
	Root_Bacteria_Proteobacteria_Alphaproteobacteria_Rhizobiales_Rhizobiaceae_Rhizobium	9.21E-01	1.76E-02	9.62E-02	6.73E-02
	Root_Bacteria_Proteobacteria_Alphaproteobacteria_Sphingomonadales_Sphingomonadaceae_Unclassified	7.03E-01	4.63E-01	8.29E-02	4.40E-02
	Root_Bacteria_Proteobacteria_Betaproteobacteria_Burkholderiales_Burkholderiaceae_Cupriavidus	2.37E-01	7.87E-01	5.53E-02	4.52E-02
	Root_Bacteria_Proteobacteria_Deltaproteobacteria_Desulfovibrionales_Desulfovibrionaceae_Bilophila	6.81E-01	2.57E-01	1.89E-01	4.90E-02
	Root_Bacteria_Proteobacteria_Gammaproteobacteria_Enterobacteriales_Enterobacteriaceae_Shigella	5.42E-01	1.70E-01	1.05E-01	5.52E-04
	Root_Bacteria_Proteobacteria_Gammaproteobacteria_Enterobacteriales_Enterobacteriaceae_Unclassified	3.39E-01	2.37E-01	2.12E-02	4.31E-04
	Root_Bacteria_Proteobacteria_Gammaproteobacteria_Unclassified	1.27E-01	1.39E-01	1.38E-02	6.57E-05
	Root_Bacteria_Proteobacteria_Unclassified	9.00E-03	1.95E-01	2.96E-01	1.06E-02
	Root_Bacteria_Unclassified	6.43E-01	4.54E-01	3.10E-02	4.58E-02
