# Supplementary material for: Association of gut microbiota with post-operative clinical course in Crohn’s disease
Source: BMC Gastroenterol. 2013 Aug 22;13:131. doi: 10.1186/1471-230X-13-131 (PMC3848607; doi:10.1186/1471-230X-13-131)
Supplement: Additional file 2: Figure S1 — Average weighted UniFrac distances (±s.e.m.) among subsets of biopsies. Asterisks indicate significant differences: * P<0.05; ** P<0.005 (Student’s t-test with 1,000 Monte Carlo simulations). Figure S2. PCoA plot of weighted UniFrac distances (A) between all samples acquired in this study (stool is indicated with an asterisk) and (B) between stool samples and biopsies acquired at the same time in 5 patients with Crohn’s disease, at the first 1–2 postoperative colonoscopies. Figure S3. PCoA plot of weighted UniFrac distances between all biopsies (i.e., from all patients at all time points) denoting samples from Crohn’s patients who underwent surgery but were excluded from predictive modeling. 1 patient was lost to follow-up. The remaining 4 underwent post-op colonoscopy and were given Rutgeerts scores of 0, 1, 2, and 3, referred to as patients 0–3, respectively, in this discussion. Patient 0 was scheduled for ileocolic resection when recruited to this study. However, an intra-operative decision was made to perform a limited ileal resection combined with stricturoplasty. During postop colonoscopy, the scope could not be advanced to the surgical anastomosis, and so the Rutgeerts score (which was based on the visualized terminal ileum) was not reflective of mucosal health at the anastomosis. Patient 1 agreed to provide surgical specimens but declined to provide biopsies at post-op colonoscopy. Patient 2 had postop colonoscopy after the sample collection period had ended. Patient 3 received postop colonoscopy at another hospital but was described to have significant inflammation and narrowing at the anastomosis, consistent with a score of 3. Figure S4. Average weighted UniFrac distances (±s.e.m.) (A) between Crohn’s surgical and all control biopsies, and (B) among surgical biopsies from an individual Crohn’s patient, demonstrating differences in remission versus recurrence. The asterisk indicates a significant difference: * P<0.05; ns, non-significant. Figure S5. Leave-o [file 1471-230X-13-131-S2.pdf]

Supplementary material for "Association of Gut Microbiota with Post-operative Clinical Course in Crohn's Disease"

Neelendu Dey, David AW Soergel, Susanna Repo, Steven E Brenner

Methods

Human subjects (additional details). Patients were asked to complete a questionnaire regarding age, gender, ethnicity, place of birth, travel history, elements of the Harvey-Bradshaw index, past medical/surgical history including year of Crohn's diagnosis (if applicable), and medication history (including antibiotics and probiotics) (Table S1). Underlying medical conditions of non-IBD controls included heart disease (2 individuals) and hepatitis B (1 individual); subjects were otherwise healthy. 11 patients with ileal Crohn's disease scheduled to undergo ileocolic resection agreed to participate; 10 ultimately underwent ileocolic resection. The 11th patient received a limited ileal resection combined with stricturoplasty (an intra-operative decision); the anastomosis could not be fully examined during colonoscopy, and he was thus excluded from subsequent analysis. 4 others were additionally excluded: 1 patient declined to contribute biopsies at colonoscopy; 1 patient received post-operative colonoscopy at another hospital; 1 patient received post-operative colonoscopy after the study period closed; and 1 patient was lost to follow-up. Rutgeerts scores at post-operative colonoscopy for the remaining 6 patients were as follows: "0-1" (herein referred to as "0"), 1, 1, 2, 2, and 2. A Rutgeerts score of 2 or more indicates recurrence; less than 2, remission. We additionally obtained samples from 9 patients without inflammatory bowel disease (IBD) undergoing ileocolic resection and 26 patients receiving only colonoscopy (15 with Crohn's disease, 11 without IBD). In the main text, the term "controls" refers to patients without IBD; those undergoing surgery are referred to as "surgical controls." Two patients declined participation altogether (one Crohn's patient prior to surgery, one control patient prior to surgery).

Sample collection (surgery). Surgeries were performed as per norm. Following surgical resection, the specimen was placed on the sterile table and dissected carefully

using sterile forceps and scissors. Samples were taken with care to minimize the area of contact with surgical tools. Ileum was sampled before colon, and within each location, inflamed mucosa was biopsied before healthy-appearing mucosa. Biopsies were placed within sterile CryoVial tubes, snap-frozen in pre-cooled methyl-butane in dry ice, and stored at -80°C until use.

Sample collection (colonoscopy). Colonoscopies were performed as per norm. The Rutgeerts score assigned by the gastroenterologist following inspection of the neo-terminal ileum and anastomosis was recorded. Endoscopic biopsies were then taken via a single channel of the colonoscope using standard biopsy forceps [1]. Ileal biopsies were taken approximately 10 cm proximal to the ileocecal valve or ileocolic anastomosis, and colonic biopsies were taken 10 cm distal to these landmarks. Ileum was sampled before colon, and within each location, inflamed mucosa was biopsied before healthy-appearing mucosa. Biopsies were placed within sterile CryoVial tubes, snap-frozen in pre-cooled methyl-butane in dry ice, and stored at -80°C until use.

Sample collection (stool). Patients who consented to participate in this study prior to surgery were contacted for stool samples just prior to post-operative colonoscopy. Patients were given a sterile stool sample collection container and sterile tongue depressors for sample acquisition. Stool samples were collected as soon as possible, placed within sterile CryoVial tubes, snap-frozen in pre-cooled methyl-butane in dry ice, and stored at -80°C until use.

Estimation of OTU count inflation due to sequencing error. We simulated the effects of noise and deep sequencing on OTU counts using repeated sampling of previously published datasets of near-full-length 16S ribosomal sequences of human gut microbial communities [2, 3]. We first extracted 101 base pair reads from the end of primer 529R, matching our experimental design. The resulting partial 16S ribosomal datasets were clustered at 97% sequence identity using UCLUST [4] with options "--iddef 2 --maxrejects 128 --nowordcountreject". We then sampled uniformly with

replacement from each fragment dataset, adding noise to each sampled read at a rate of 0.1% mismatches per base with no indels, to produce simulated datasets of 200 million reads each, matching the size of our combined dataset. Finally, these large simulated datasets were clustered at 97% sequence identity using UCLUST. Conversely, we investigated the impact of shallower sequencing in our study on OTU counts by subsampling 10,000 sequences per sample (performing 5 replicates), clustering the results using UCLUST as previously, and comparing again to the same prior datasets [2, 3].

References

1. Dave M, Johnson LA, Walk ST, Young VB, Stidham RW, Chaudhary MN, Funnell J, Higgins PDR: A randomised trial of sheathed versus standard forceps for obtaining uncontaminated biopsy specimens of microbiota from the terminal ileum. *Gut* 2011.

2. Turnbaugh PJ, Hamady M, Yatsunenko T, Cantarel BL, Duncan A, Ley RE, Sogin ML, Jones WJ, Roe BA, Affourtit JP, Egholm M, Henrissat B, Heath AC, Knight R, Gordon JI: A core gut microbiome in obese and lean twins. *Nature* 2009, 457:480–484.

3. Dethlefsen L, Huse S, Sogin ML, Relman DA: The pervasive effects of an antibiotic on the human gut microbiota, as revealed by deep 16S rRNA sequencing. *PLoS Biol* 2008, 6:e280.

4. Edgar RC: Search and clustering orders of magnitude faster than BLAST. *Bioinformatics* 2010.

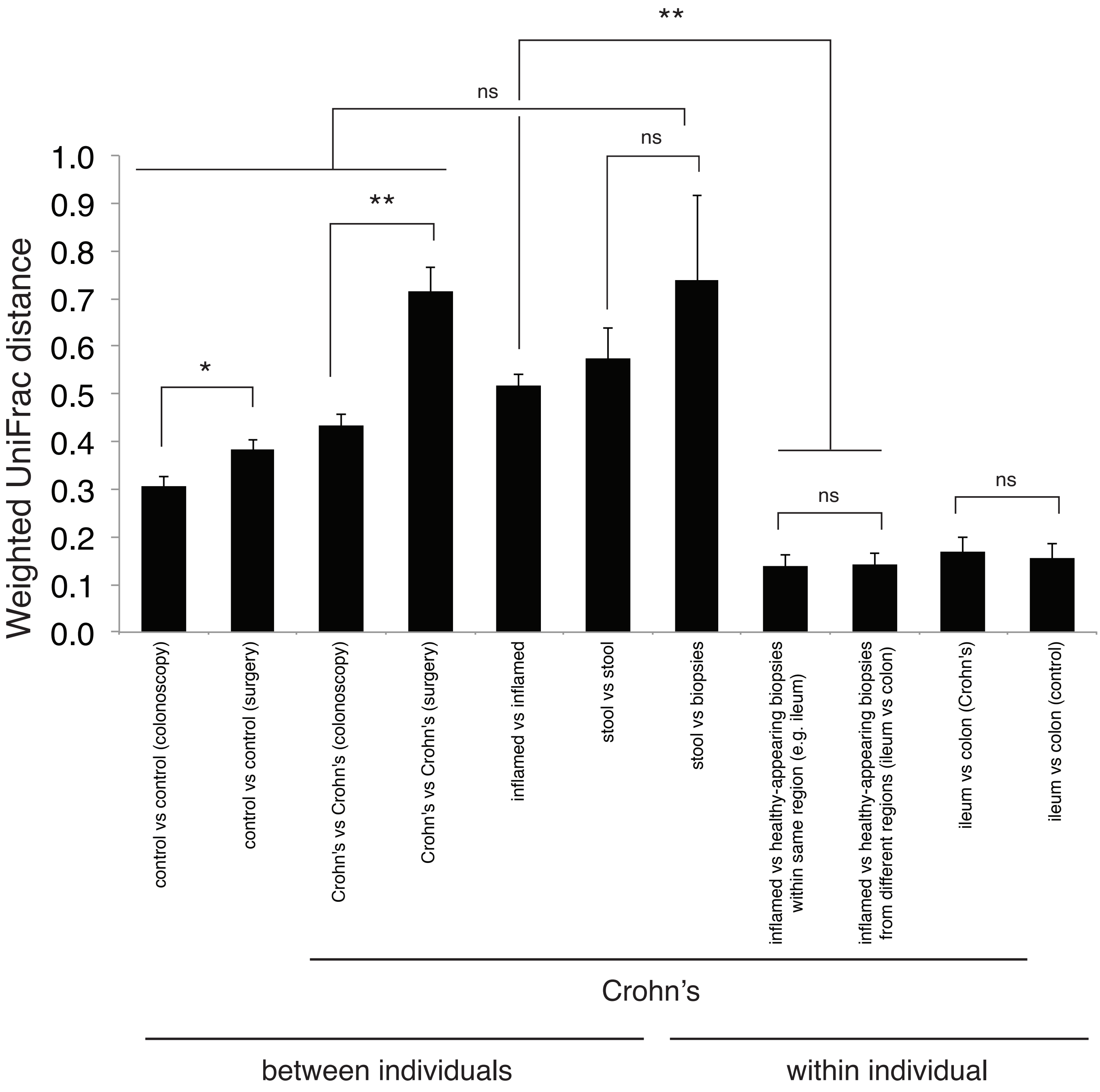

Fig. S1.

Average weighted UniFrac distances (±s.e.m.) among subsets of biopsies. Asterisks indicate significant differences: * $P<0.05$; ** $P<0.005$ (Student’s *t*-test with 1,000 Monte Carlo simulations).

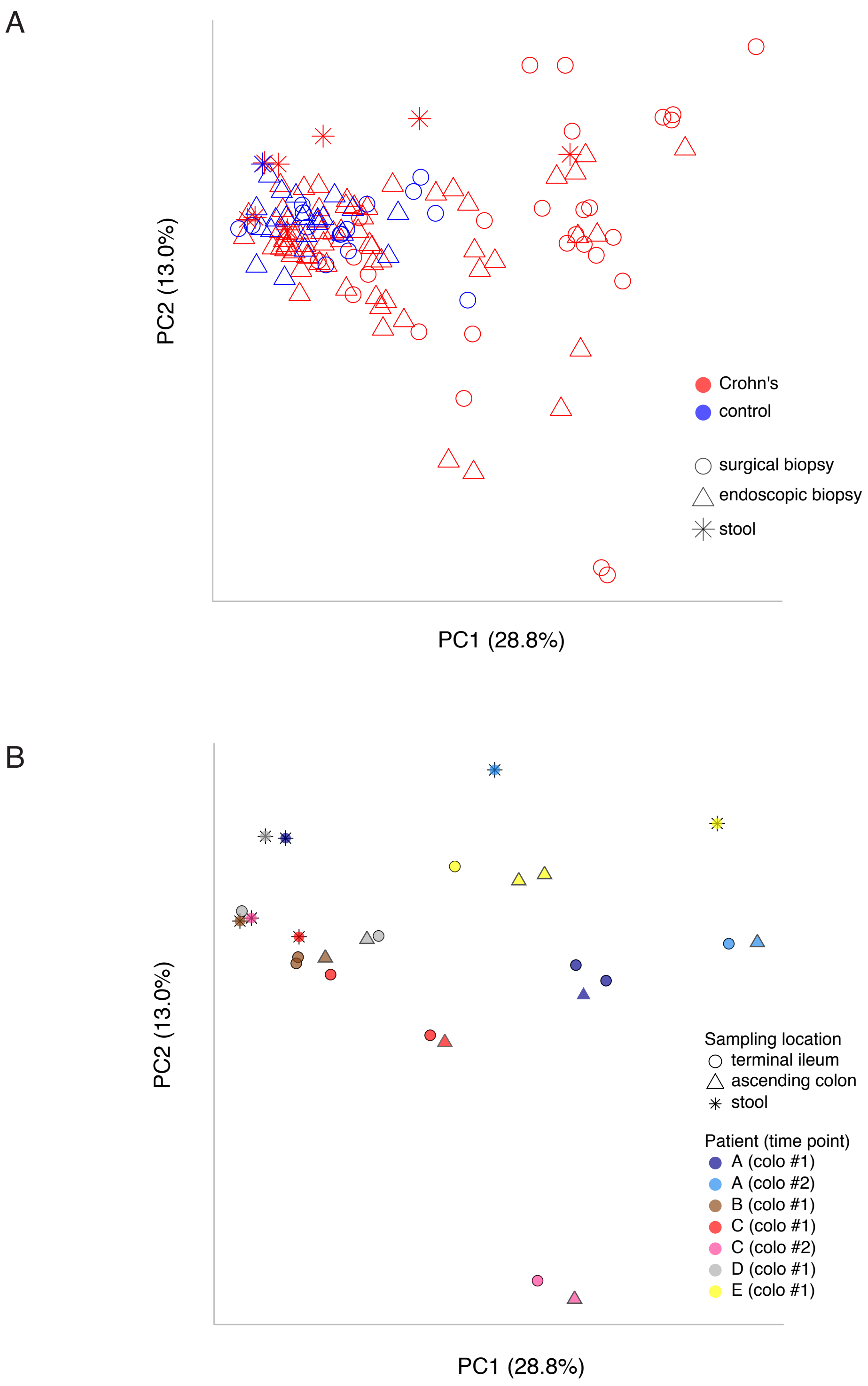

Fig. S2.
PCoA plot of weighted UniFrac distances (*A*) between all samples acquired in this study (stool is indicated with an asterisk) and (*B*) between stool samples and biopsies acquired at the same time in 5 patients with Crohn's disease, at the first 1-2 post-operative colonoscopies.

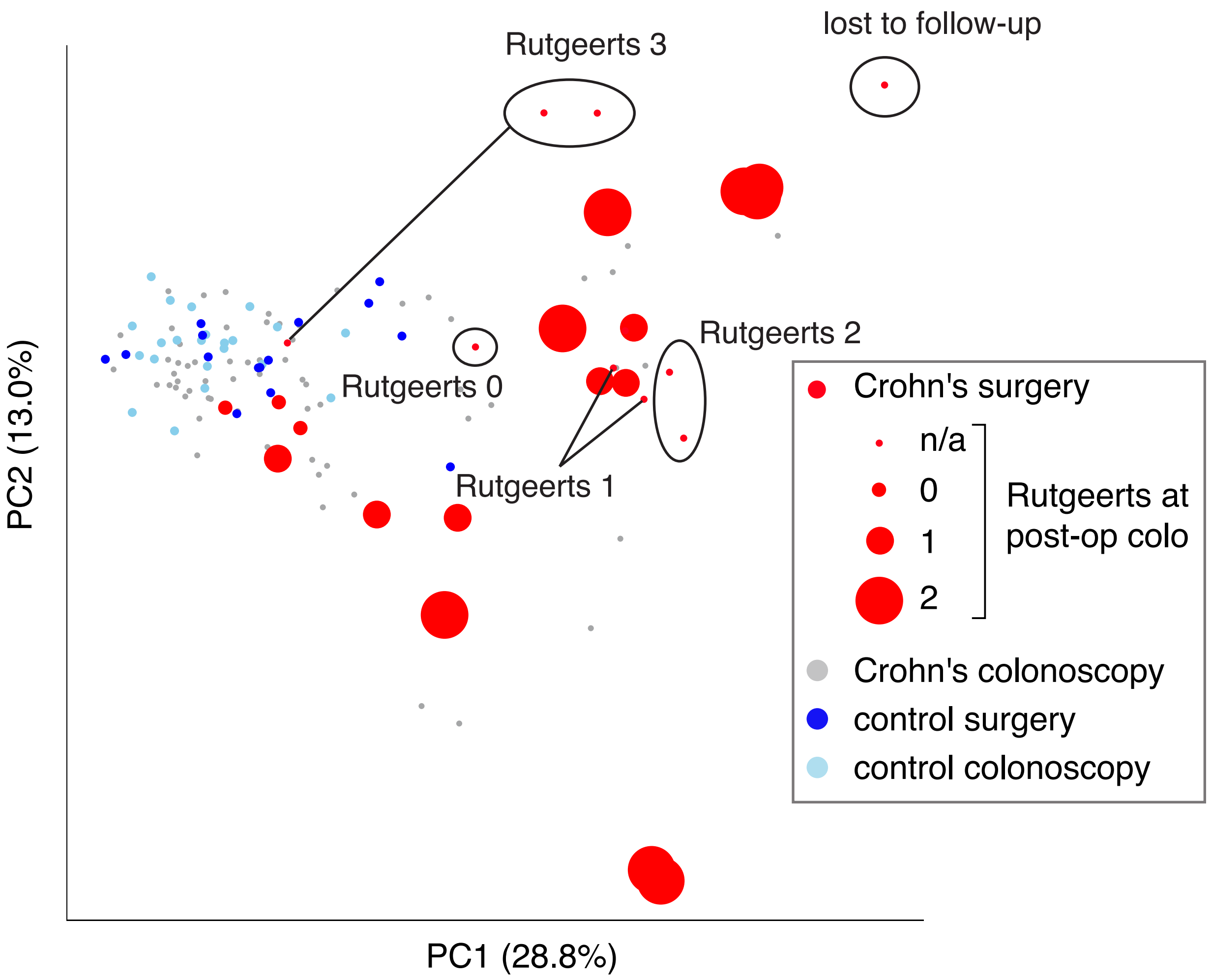

Fig. S3.

PCoA plot of weighted UniFrac distances between all biopsies (i.e., from all patients at all time points) denoting samples from Crohn's patients who underwent surgery but were excluded from predictive modeling. 1 patient was lost to follow-up. The remaining 4 underwent post-op colonoscopy and were given Rutgeerts scores of 0, 1, 2, and 3, referred to as patients 0-3, respectively, in this discussion. Patient 0 was scheduled for ileocolic resection when recruited to this study. However, an intra-operative decision was made to perform a limited ileal resection combined with stricturoplasty. During post-op colonoscopy, the scope could not be advanced to the surgical anastomosis, and so the Rutgeerts score (which was based on the visualized terminal ileum) was not reflective of mucosal health at the anastomosis. Patient 1 agreed to provide surgical specimens but declined to provide biopsies at post-op colonoscopy. Patient 2 had post-op colonoscopy after the sample collection period had ended. Patient 3 received post-op colonoscopy at another hospital but was described to have significant inflammation and narrowing at the anastomosis, consistent with a score of 3.

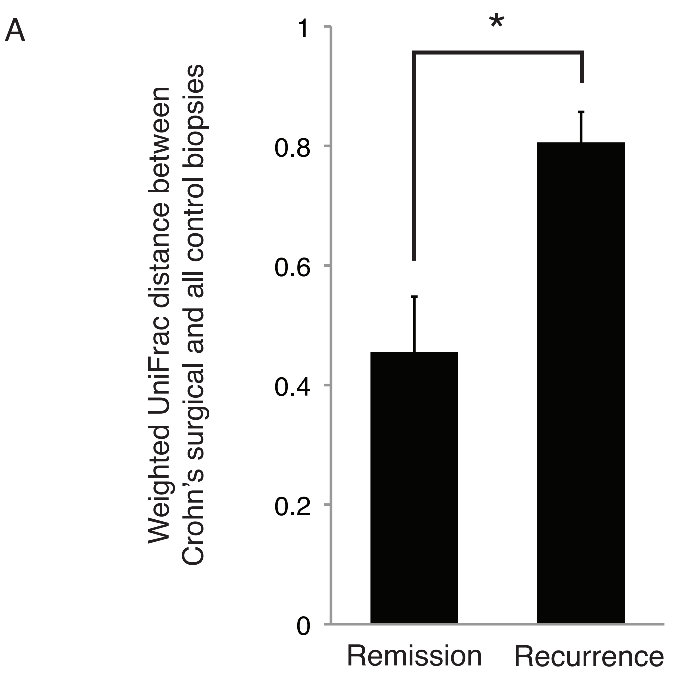

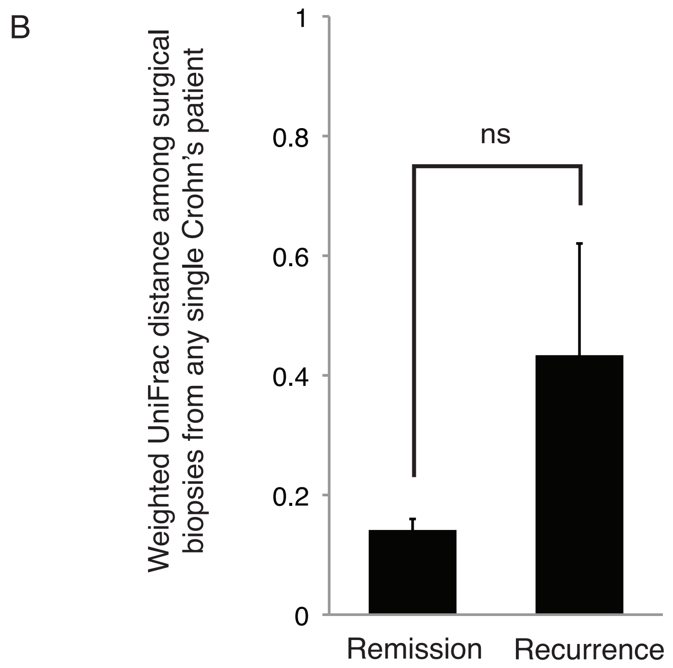

Fig. S4.

Average weighted UniFrac distances (±s.e.m.) (*A*) between Crohn's surgical and all control biopsies, and (*B*) among surgical biopsies from an individual Crohn's patient, demonstrating differences in remission versus recurrence. The asterisk indicates a significant difference: * $P<0.05$; ns, non-significant.

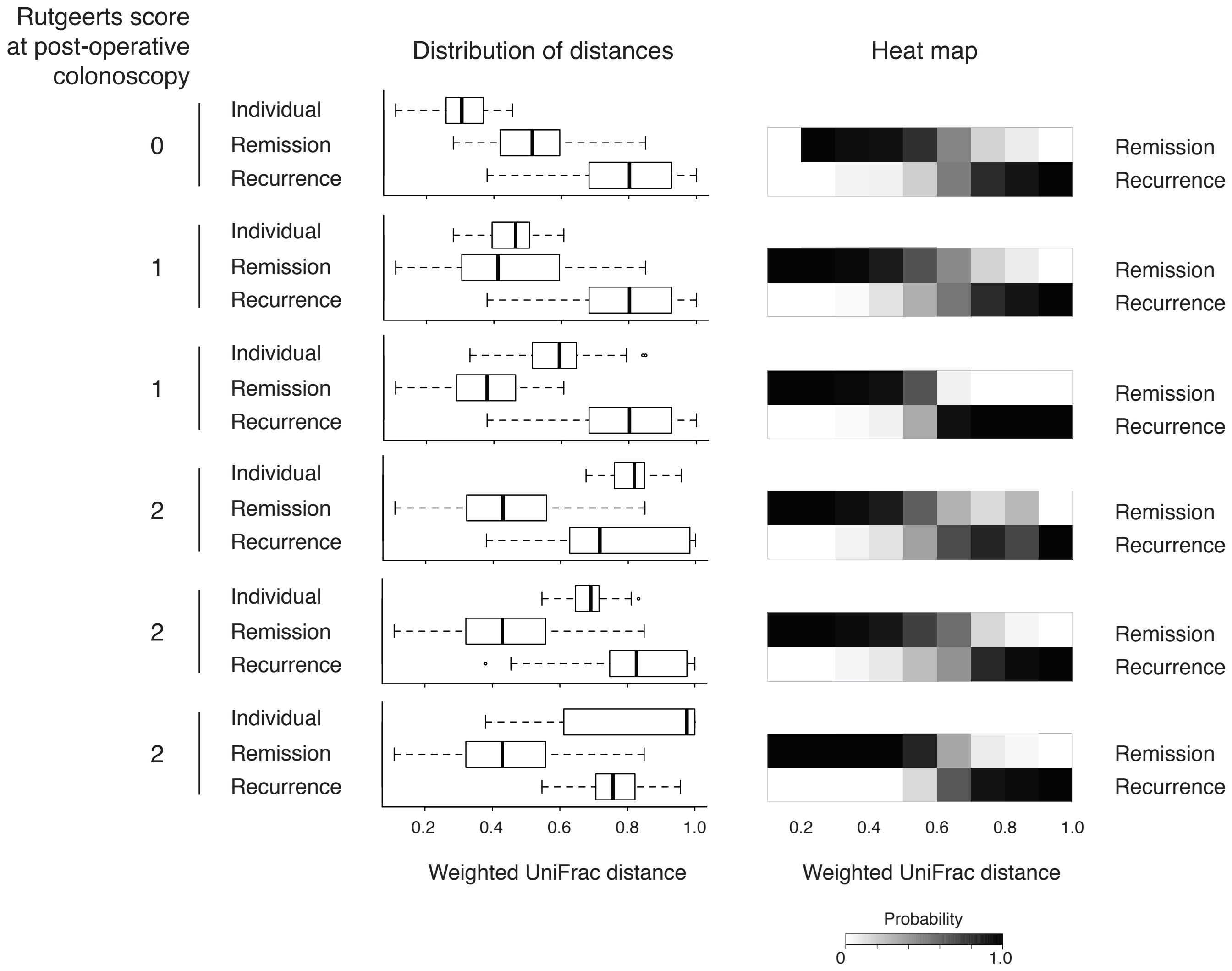

Fig. S5.

Leave-one-out validation testing of heat maps for generating predictions regarding post-operative recurrence in Crohn's patients using weighted UniFrac distances between biopsies taken at surgery and surgical controls. The post-operative Rutgeerts score is shown on the left (remission = 1-0; recurrence = 2 or more). The distribution of UniFrac distances for each patient is represented as the top box plot in each set of three. The subsequent two box plots in each set are the distributions of UniFrac distances in the remaining 5 patients, distinguishing those who had recurrence from those in remission. Heat maps on the right were generated from these distributions to reflect probabilities of recurrence or remission given a single UniFrac distance.

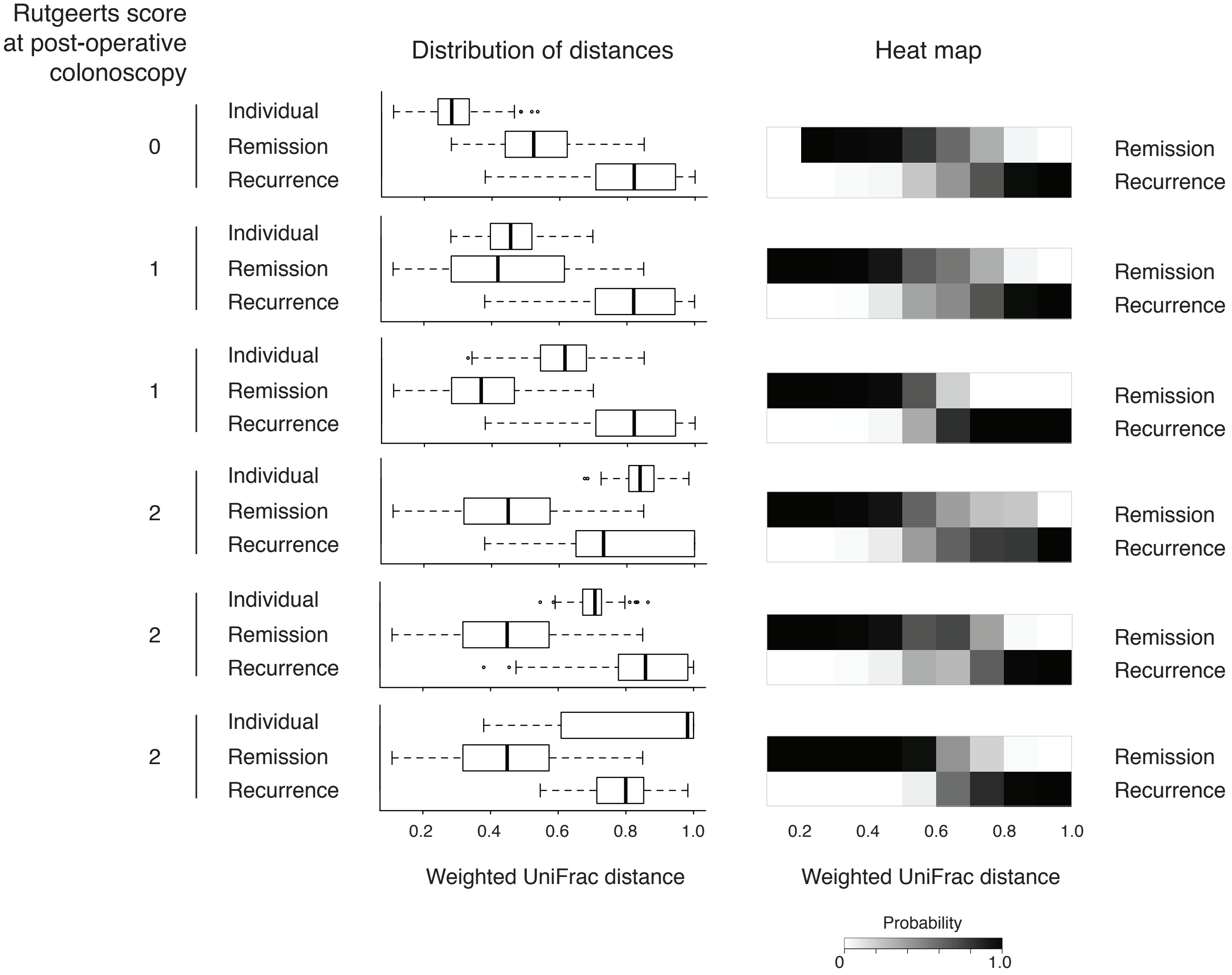

Fig. S6.

Leave-one-out validation testing of heat maps for generating predictions regarding post-operative recurrence in Crohn's patients using weighted UniFrac distances between biopsies taken at surgery and all control biopsies (taken from surgery and colonoscopy). The post-operative Rutgeerts score is shown on the left (remission = 0-1; remission = 2 or more). The distribution of UniFrac distances for each patient is represented as the top box plot in each set of three. The subsequent two box plots in each set are the distributions of UniFrac distances in the remaining 5 patients, distinguishing those who had recurrence from those in remission. Heat maps on the right were generated from these distributions to reflect probabilities of recurrence or remission given a single UniFrac distance.

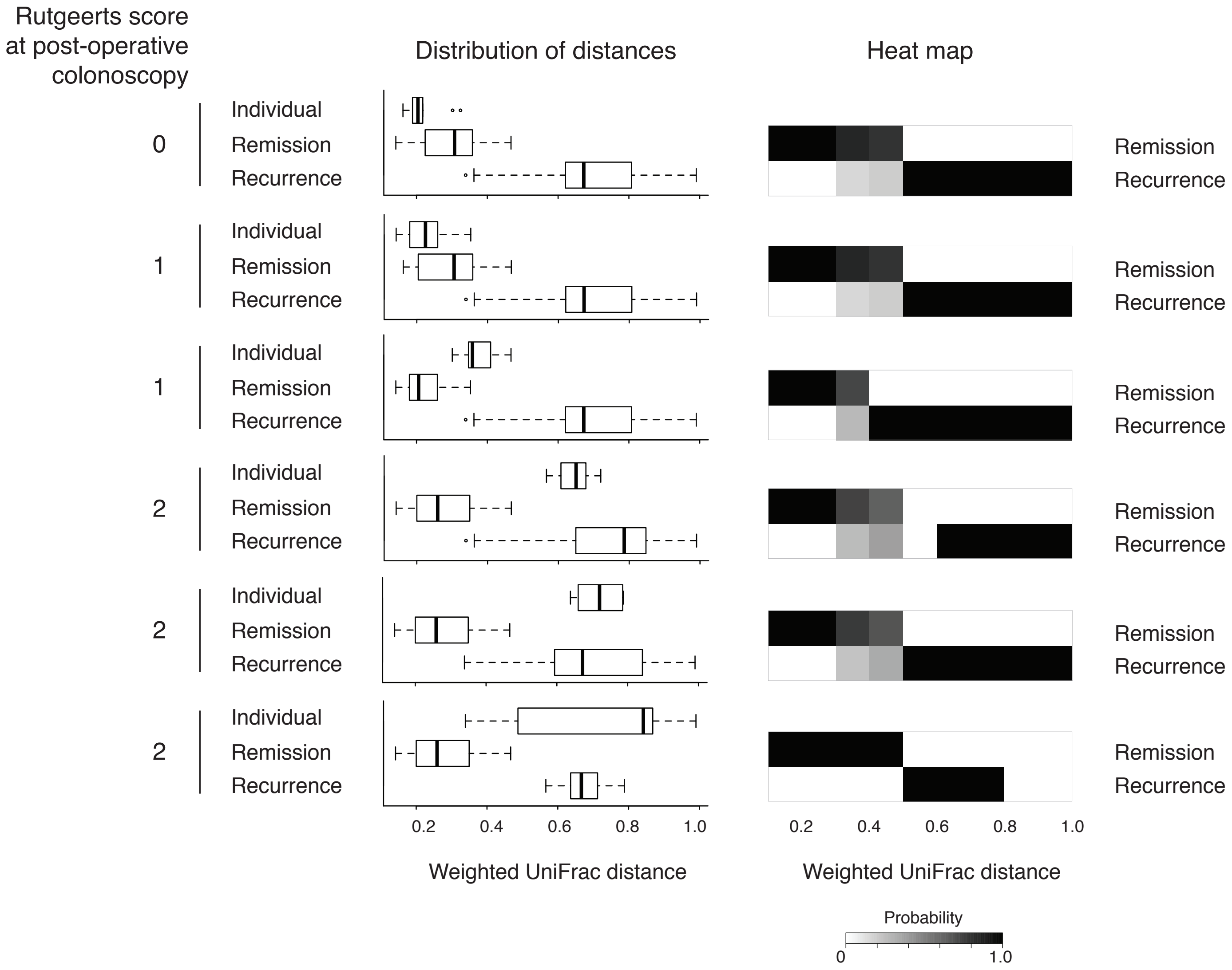

Fig. S7.

Leave-one-out validation testing of heat maps for generating predictions regarding post-operative recurrence in Crohn's patients using weighted UniFrac distances between biopsies taken at surgery and at post-operative colonoscopy. The post-operative Rutgeerts score is shown on the left (remission = 0-1; remission = 2 or more). The distribution of UniFrac distances for each patient is represented as the top box plot in each set of three. The subsequent two box plots in each set are the distributions of UniFrac distances in the remaining 5 patients, distinguishing those who had recurrence from those in remission. Heat maps on the right were generated from these distributions to reflect probabilities of recurrence or remission given a single UniFrac distance.

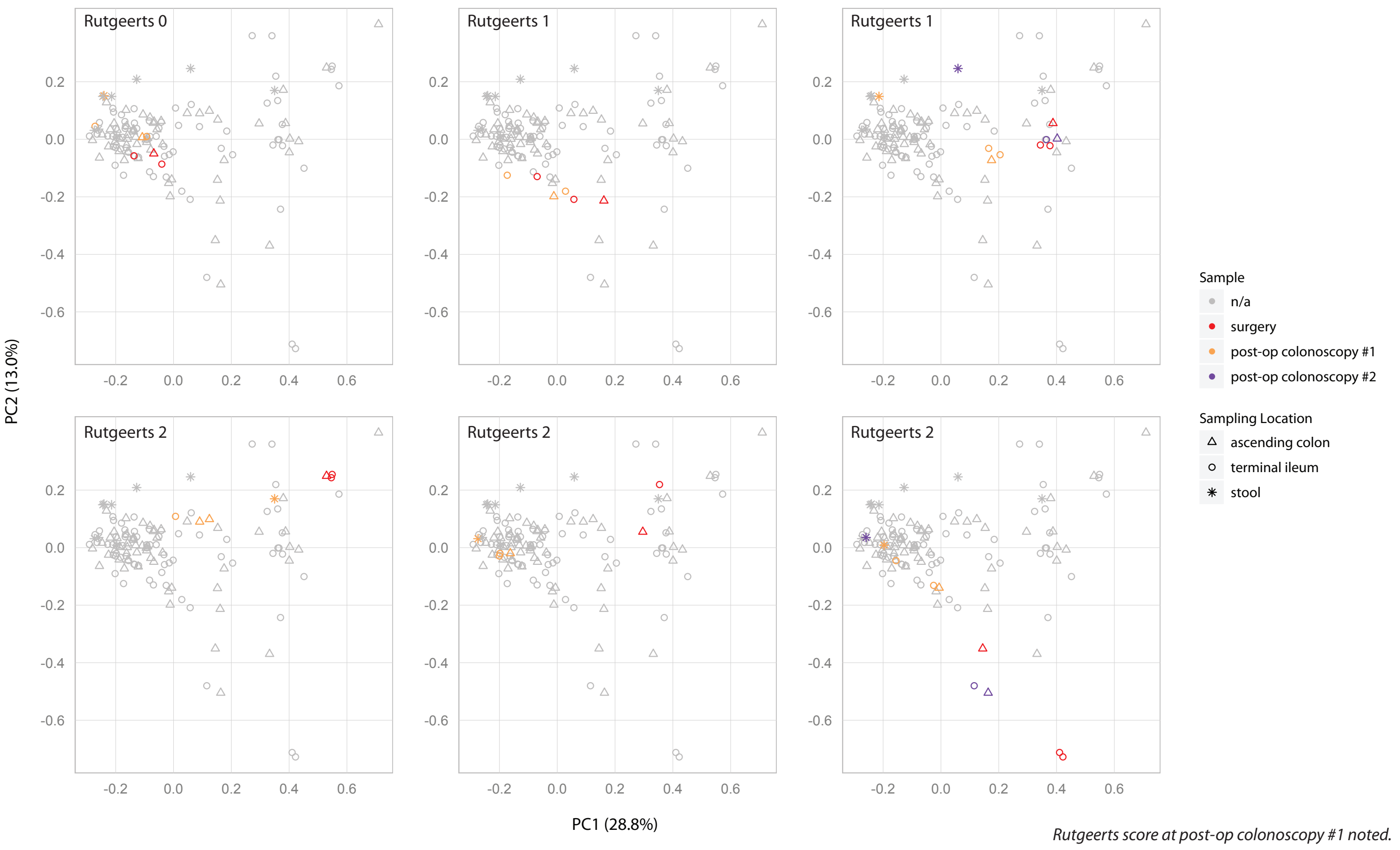

Fig. S8.

PCoA plots of weighted UniFrac distances between all samples acquired in this study, with each panel highlighting the progression through time of an individual patient. The top row shows patients who went into remission (Rutgeerts scores of 0 or 1); the bottom row shows patients who had recurrence (Rutgeerts scores of 2).

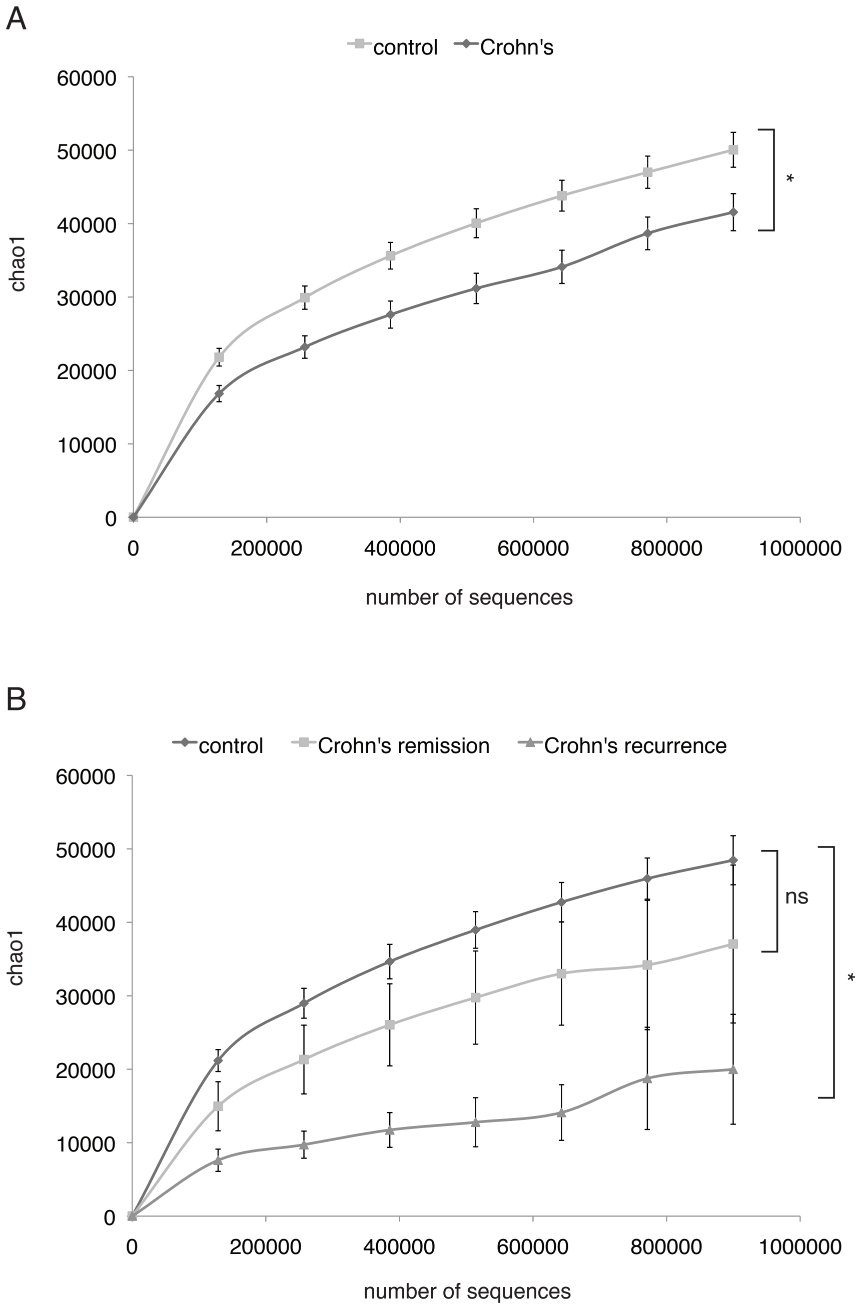

Fig. S9.

Alpha diversity as measured using the chao1 metric, comparing (*A*) control versus Crohn's samples and (*B*) surgical biopsies of Crohn's patients who go on to have recurrence versus those who stay in remission, compared to surgical control biopsies. The asterisk indicates a significant difference: * $P<0.05$.

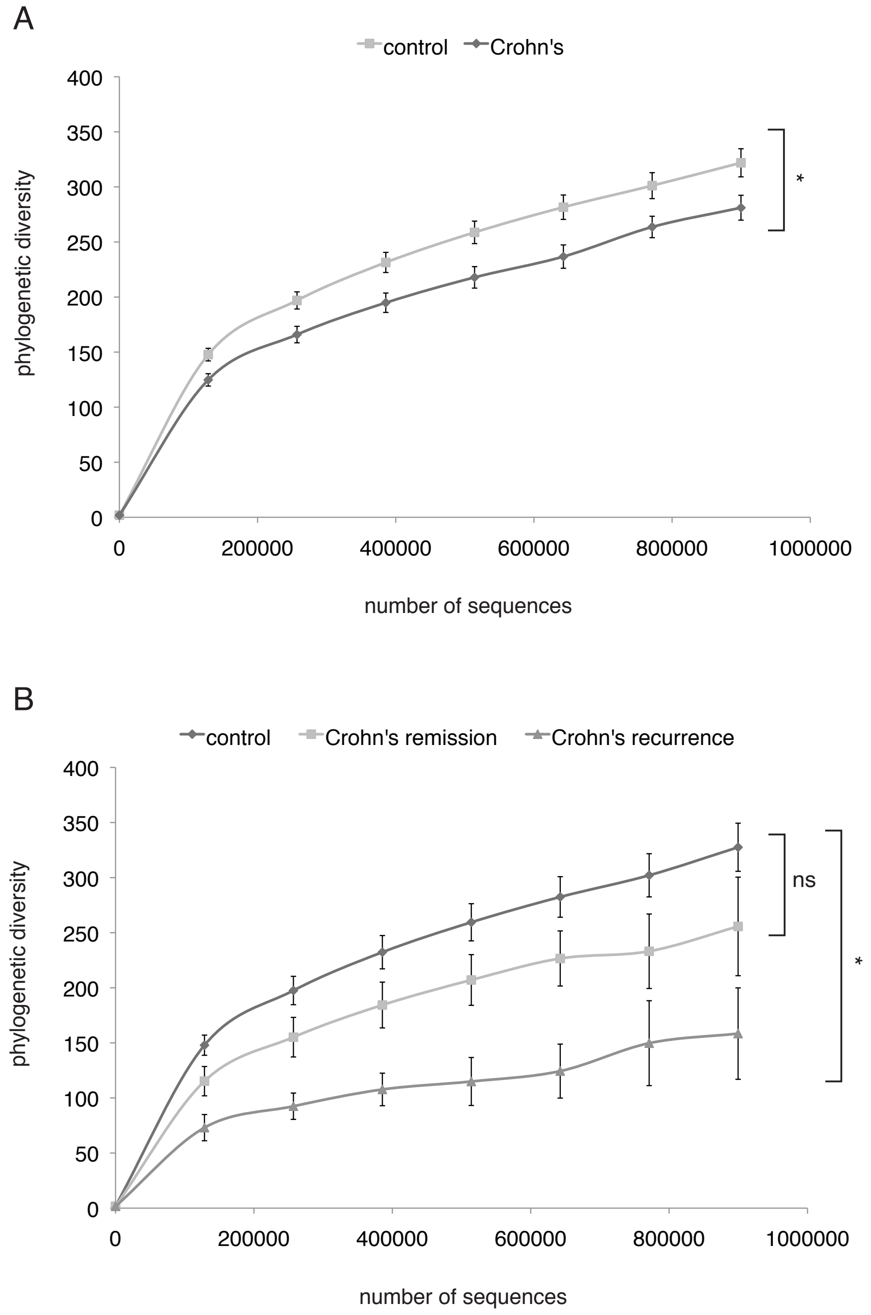

Fig. S10.

Alpha diversity as measured using phylogenetic diversity, comparing (*A*) control versus Crohn's samples and (*B*) surgical biopsies of Crohn's patients who go on to have recurrence versus those who stay in remission, compared to surgical control biopsies. The asterisk indicates a significant difference: * $P<0.05$.
